# JAG1-NOTCH4 mechanosensing drives atherosclerosis

**DOI:** 10.1126/sciadv.abo7958

**Published:** 2022-08-31

**Authors:** Celine Souilhol, Blanca Tardajos Ayllon, Xiuying Li, Mannekomba R. Diagbouga, Ziqi Zhou, Lindsay Canham, Hannah Roddie, Daniela Pirri, Emily V. Chambers, Mark J. Dunning, Mark Ariaans, Jin Li, Yun Fang, Helle F. Jørgensen, Michael Simons, Rob Krams, Johannes Waltenberger, Maria Fragiadaki, Victoria Ridger, Sarah De Val, Sheila E. Francis, Timothy JA Chico, Jovana Serbanovic-Canic, Paul C. Evans

**Affiliations:** ^1^ Department of Infection, Immunity and Cardiovascular Disease, INSIGNEO Institute for In Silico Medicine, and the Bateson Centre, University of Sheffield, Sheffield, UK.; ^2^ Biomolecular Sciences Research Centre, Sheffield Hallam University, Sheffield, UK.; ^3^ School of Pharmacy, Southwest Medical University, LuZhou, Sichuan 646000, P.R. China.; ^4^ Sheffield Bioinformatics Core, Sheffield Institute of Translational Neuroscience, University of Sheffield, Sheffield, UK.; ^5^ Biological Sciences Division, Department of Medicine, University of Chicago, Chicago, IL, USA.; ^6^ Division of Cardiovascular Medicine, University of Cambridge, Addenbrooke’s Centre for Clinical Investigation, Addenbrooke’s Hospital, Cambridge, UK.; ^7^ Department of Internal Medicine, Yale Cardiovascular Research Center, New Haven, CT, USA.; ^8^ Department of Bioengineering, Queen Mary University of London, London, UK.; ^9^ Department of Cardiovascular Medicine, Medical Faculty, University of Münster, Münster, Germany.; ^10^ Hirslanden Klinik im Park, Cardiovascular Medicine, Diagnostic and Therapeutic Heart Center AG, 8002 Zürich, Switzerland.; ^11^ BHF Centre of Regenerative Medicine, Department of Physiology, Anatomy and Genetics, University of Oxford, Oxford, UK.; ^12^ Ludwig Institute for Cancer Research Ltd, Nuffield Department of Medicine, University of Oxford, Oxford OX3 7DQ, UK.

## Abstract

Endothelial cell (EC) sensing of disturbed blood flow triggers atherosclerosis, a disease of arteries that causes heart attack and stroke, through poorly defined mechanisms. The Notch pathway plays a central role in blood vessel growth and homeostasis, but its potential role in sensing of disturbed flow has not been previously studied. Here, we show using porcine and murine arteries and cultured human coronary artery EC that disturbed flow activates the JAG1-NOTCH4 signaling pathway. Light-sheet imaging revealed enrichment of JAG1 and NOTCH4 in EC of atherosclerotic plaques, and EC-specific genetic deletion of *Jag1* (*Jag1^ECKO^*) demonstrated that *Jag1* promotes atherosclerosis at sites of disturbed flow. Mechanistically, single-cell RNA sequencing in *Jag1^ECKO^* mice demonstrated that *Jag1* suppresses subsets of ECs that proliferate and migrate. We conclude that JAG1-NOTCH4 sensing of disturbed flow enhances atherosclerosis susceptibility by regulating EC heterogeneity and that therapeutic targeting of this pathway may treat atherosclerosis.

## Teaser

Complex blood flow promotes cardiovascular disease by activating a mechanosensitive pathway that suppresses arterial repair.

## INTRODUCTION

Blood flow generates mechanical shear stress that has profound effects on the function of blood vessels by altering the physiology of vascular endothelial cells (ECs) (*1*). Shear stress controls the initiation and progression of atherosclerosis, a disease characterized by the accumulation of cells, lipids, and other materials in the arterial wall, leading to angina, myocardial infarction, and stroke (*2*, *3*). Regions of the arterial tree with uniform geometry are exposed to high physiological shear stress that is unidirectional, which promotes EC health and protection from atherosclerosis. However, branches and bends of arteries are exposed to complex blood flow patterns, generating low time-averaged shear stress that varies in direction (e.g., oscillatory bidirectional and biaxial flow) (*4*). These disturbed flow conditions promote EC dysfunction and the initiation of atherosclerosis. Disturbed flow drives both angiogenesis and atherosclerosis, suggesting a common mechanism for these different vascular processes (*1*).

The Notch pathway was found in *Drosophila* as a master regulator of cell fate decisions and the spatial organization of tissues (*5*). Canonical Notch signaling involves an interaction between a Notch transmembrane receptor and a canonical Notch ligand on an adjacent cell, which causes proteolytic cleavage of the Notch receptor by γ-secretase. This causes release of the Notch intracellular domain (ICD), which localizes to the nucleus to regulate transcription (*6*). Mammals have four Notch receptors and five ligands. Classic studies revealed that the interaction between NOTCH1 and DLL4 on adjacent EC establishes “tip” versus “stalk” cell identity during angiogenesis (*7*), and NOTCH1 and DLL4 are critical for arterial specification (*8*–*10*) by suppressing EC proliferation (*11*) and neovascularization in ischemic tissues (*12*, *13*). Notch activation requires mechanical force induced by endocytosis of the ligand (*14*), and NOTCH1 sensing of shear stress is important in arterial differentiation (*15*) and vascular homeostasis (*16*) and protection (*17*). However, the potential role of the Notch system in EC sensing of proatherosclerotic disturbed flow has not been previously studied.

Here, we used several complementary experimental approaches (porcine arteries, experimental flow–modified mouse arteries, and cultured human EC) to show that disturbed flow primarily activates the JAG1-NOTCH4 pathway. Conditional genetic deletion demonstrated that *Jag1* enhances atherosclerosis specifically at regions of disturbed flow that are prone to disease. Single-cell RNA sequencing (scRNA-seq) and other mechanistic approaches showed that JAG1-NOTCH4 signaling promotes atherosclerosis by repressing EC subsets that are critical for proliferation. These data fundamentally advance our knowledge of the role of Notch signaling in interpreting shear stress signals to control EC function and have important implications for therapeutic targeting of the Notch pathway in atherosclerosis.

## RESULTS

### JAG1 and NOTCH4 are enriched at atherosusceptible regions exposed to disturbed flow

**Fig. 1.**
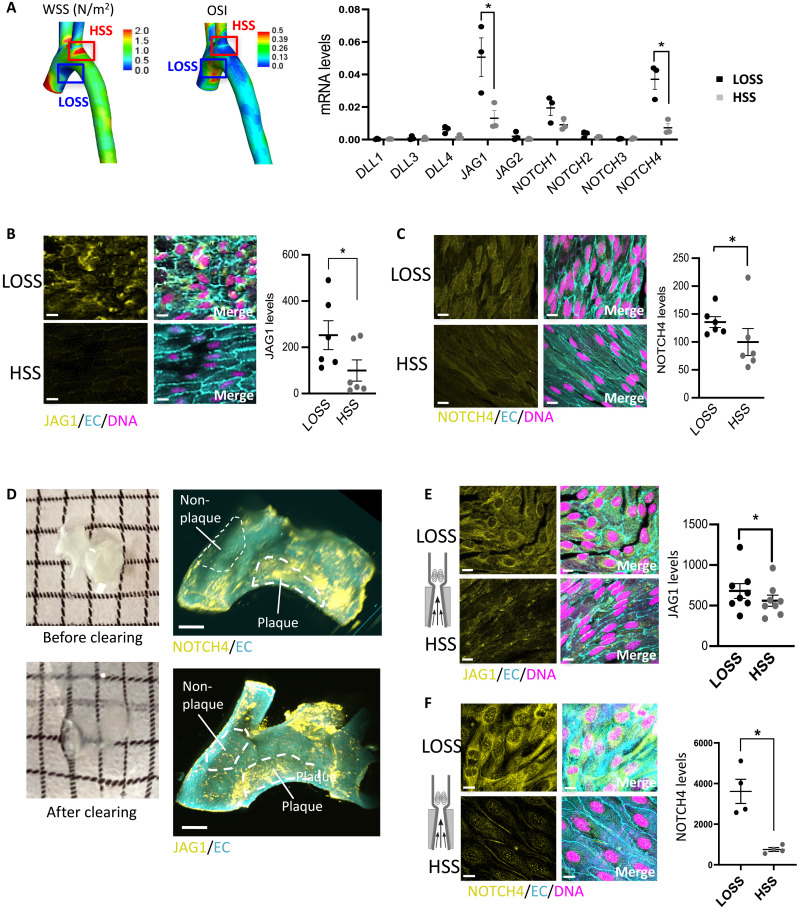
JAG1 and NOTCH4 are enriched at atheroprone sites. (**A**) ECs were isolated from LOSS versus HSS regions of the porcine aorta based on models of time-averaged wall shear stress (WSS) (left) and oscillatory shear index (OSI; center). Shear stress maps were adapted from (*18*). The expression of Notch receptors and ligands was quantified in each population by qRT-PCR (*n* = 3). (**B** and **C**) Aortic arches were isolated from C57BL/6 mice, and en face immunostainings were performed using anti-JAG1 (B) or anti-NOTCH4 (C) antibodies (yellow). Endothelium was costained (anti-CDH5 or anti-CD31; EC; cyan), and nuclei were detected using TO-PRO-3 (DNA; magenta). The graphs on the right represent the yellow mean fluorescence intensity (*n* = 6). (**D**) Aortic arches were isolated from ApoE^−/−^ mice exposed to a high-fat diet for 6 weeks. Samples were optically cleared before immunofluorescence staining using anti-NOTCH4 (top) or anti-JAG1 (bottom) antibodies (yellow). Endothelium was costained (anti-CDH5; EC; cyan). Representative light-sheet images are shown with plaque areas and nonplaque areas delineated. (**E** and **F**) Flow-altering, constrictive cuffs were placed on the right carotid arteries of C57BL/6 mice. They generated anatomically distinct regions exposed to HSS and LOSS. Carotid arteries were harvested after 14 days, and en face staining was performed using anti-JAG1 (E) or anti-NOTCH4 (F) antibodies (yellow). Endothelium was costained (anti-CDH5; EC; cyan), and nuclei were detected using TO-PRO-3 (DNA; magenta). Representative images and quantification of JAG1 (*n* = 8 mice) or NOTCH4 (*n* = 4 mice) expression are shown. Differences between means were analyzed using paired *t* tests. Scale bars, 10 μm.

We hypothesized that Notch receptors and ligands may have a different spatial pattern of expression in arterial locations exposed to different flow conditions. This was tested by quantitative reverse transcription polymerase chain reaction (qRT-PCR) analysis of the transcripts of Notch receptors and their ligands in EC isolated from regions of the porcine aorta exposed to disturbed low oscillatory shear stress (LOSS; inner curvature) or high shear stress (HSS; outer curvature) using shear stress maps generated previously by our group (*18*). We validated the approach by demonstrating enrichment of the HSS-induced gene *eNOS* at the outer curvature and enrichment of the LOSS-induced inflammatory gene *MCP-1* at the inner curvature (fig. S1). The expression of *JAG1* and *NOTCH4* mRNA was significantly enhanced at sites of LOSS compared to HSS, whereas the expression of *JAG2*, *DLL1*, *DLL3*, *DLL4*, *NOTCH1*, *NOTCH2*, and *NOTCH3* was similar between these sites (Fig. 1A).

We next quantified the expression of JAG1 and NOTCH4 proteins in the murine aortic arch. This region was selected because it has been spatially mapped for lesion development (*19*) and fluid dynamics (*20*), revealing that the inner curvature of the arch is susceptible to atherosclerosis and exposed to LOSS, whereas the outer curvature is protected and exposed to HSS that is more uniform in direction. Moreover, focusing on EC within the arch allows spatial differences to be uncoupled from developmental origins, which vary between the arch and other parts of the aorta (*21*). En face staining revealed that JAG1 and NOTCH4 proteins were expressed at higher levels at the LOSS region compared to the HSS region in the murine aortic arch (Fig. 1, B and C).

Analysis of diseased arteries from hypercholesterolemic apolipoprotein E (*ApoE*)^−/−^ mice using immunofluorescent light-sheet imaging revealed that JAG1 and NOTCH4 were enriched in regions of atherosclerotic plaque compared to adjacent nonplaque areas, indicating a potential role in atherosclerosis (Fig. 1D). En face staining confirmed that NOTCH4 enrichment in the plaque areas correlates with the expression in EC (fig. S2). We conclude that JAG1 and NOTCH4 are enriched in the endothelium at atherosusceptible sites and in atherosclerotic plaque.

### Disturbed flow is sufficient to activate the JAG1-NOTCH4 pathway

The association of increased JAG1 and NOTCH4 at sites of disturbed flow does not prove a causal link to LOSS since these sites are also exposed to potential confounders such as mass transport, altered oxygen levels, and inflammation. We therefore assessed whether JAG1 and NOTCH4 are directly shear stress sensitive using a constrictive cuff to modify flow in the murine carotid artery. The cuff generates HSS at the stenosis and a pulsatile vortex that induces oscillations in velocity direction downstream of the cuff (*22*, *23*). Immunofluorescent staining and confocal microscopy showed that JAG1 and NOTCH4 were significantly enhanced at the downstream region compared to the stenosis region after cuff placement for 14 days (Fig. 1, E and F). By contrast, JAG1 and NOTCH4 expression was virtually undetectable in contralateral arteries with physiological flow (fig. S3). At 14 days of cuff placement, there were no signs of inflammation. In addition to the generation of oscillatory shear stress, the pulsatile vortex generated by the cuff also changes mass transport, by trapping molecules and particles, but in a largely different way compared to the inner part of the aortic arch, which is exposed to vortex shedding and therefore intermittent changes in mass transport. Hence, JAG1 and NOTCH4 enrichment at the inner curvature and the downstream region of the cuff is likely a response to LOSS.

**Fig. 2.**
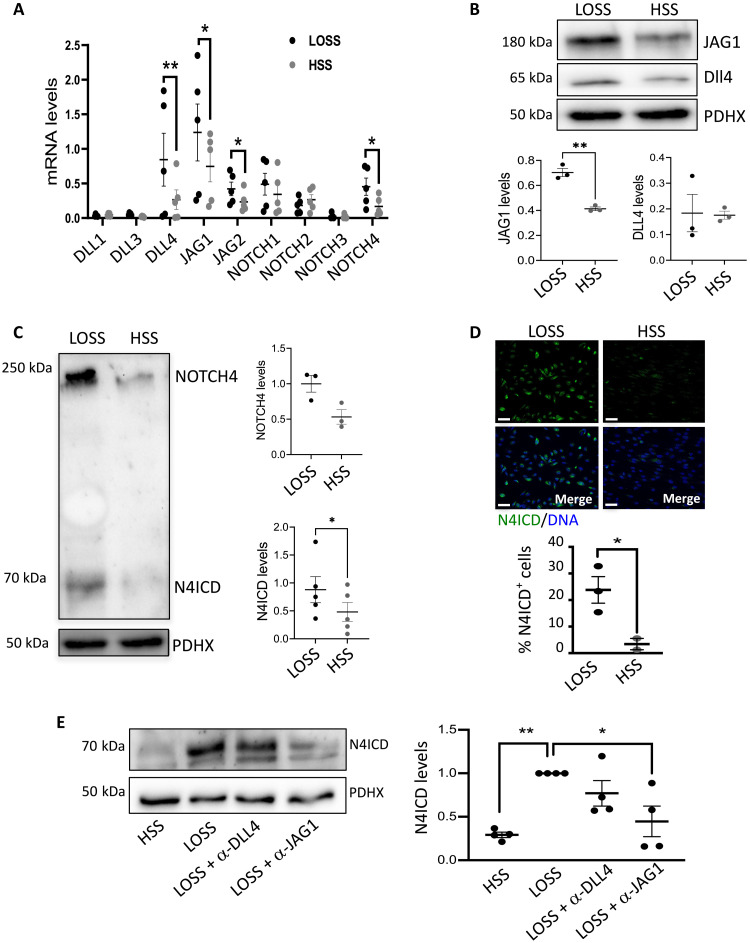
JAG1-NOTCH4 pathway is induced by LOSS in vitro. HCAECs from individual donors were seeded on μ-slides and cultured under LOSS or HSS for 72 hours using the Ibidi system. (**A**) Expression levels of Notch receptors and ligands were quantified by qRT-PCR (*n* = 5). Protein levels of JAG1 and DLL4 (**B**) or levels of total NOTCH4 and the activated forms of NOTCH4 (N4ICD) (**C**) were quantified by immunoblotting with normalization to the level of pyruvate dehydrogenase complex component X (PDHX) (*n* = 3 or *n* = 5). Representative images are shown. (**D**) Protein levels of N4ICD were quantified by immunofluorescence staining with costaining of nuclei using TO-PRO-3 (DNA). Representative images are shown, and the percentage of N4ICD-positive nuclei was calculated (*n* = 3). Scale bars, 50 μm. (**E**) HCAECs were cultured under LOSS or HSS for 72 hours using the ibidi system in the presence or absence of blocking antibodies against JAG1 or DLL4. Protein levels of N4ICD were analyzed by immunoblotting and normalized to the level of PDHX (*n* = 4). Representative images are shown. Differences between means were analyzed by analysis of variance (ANOVA).

Human coronary artery EC (HCAEC) responses to flow were analyzed because pathological changes in these cells are pivotal drivers of atherosclerotic coronary artery disease. This model was validated by demonstrating that inflammatory *MCP1* transcripts are enhanced in HCAEC exposed to LOSS, whereas the protective *KLF4* transcription factor (*24*) is enhanced by HSS (fig. S4). mRNA levels of the Notch ligands *JAG1*, *JAG2*, *DLL3*, and *DLL4* were significantly altered by shear stress in HCAEC, with *JAG1* exhibiting the highest expression under LOSS (Fig. 2A). However, JAG1 was the only Notch ligand consistently enhanced by LOSS at the protein level (Fig. 2B). DLL4 protein was unaltered by flow (Fig. 2B), and JAG2 protein was not detected. Of the Notch receptors, NOTCH4 was enhanced by LOSS at both mRNA (Fig. 2A) and protein (Fig. 2C) levels, and this was associated with increased signaling, evidenced by the increased active, cleaved ICDs of NOTCH4 (N4ICD) under LOSS (Fig. 2, C and D). By contrast, NOTCH1 was enhanced by HSS (fig. S5), which is consistent with previous reports (*16*, *17*). *JAG1* and *NOTCH4* mRNA levels were lower in HCAEC exposed to static conditions compared to flow (fig. S6), confirming that they are flow responsive. Overall, these data demonstrate that JAG1 and NOTCH4 are the dominant Notch components in human EC exposed to LOSS, which is consistent with our in vivo studies.

We next investigated whether JAG1 acts upstream from NOTCH4 under LOSS using published activity-blocking antibodies against JAG1 (*25*) [and comparing to published anti-DLL4 (*26*) blocking antibodies as a control]. To confirm their effectiveness, we demonstrated that anti-JAG1 and anti-DLL4 blocking antibodies can suppress Notch target gene expression (fig. S7A). In HCAEC exposed to LOSS, N4ICD activation was significantly reduced by blocking JAG1, whereas blocking of DLL4 activity had no significant effect (Fig. 2E). Total levels of *NOTCH4* mRNA were unaltered by anti-JAG1 or anti-DLL4 blocking antibodies (fig. S7B). In conclusion, our data point to JAG1 activation of NOTCH4 as a key pathway under LOSS conditions.

### Endothelial *Jag1* induces atherosclerosis at a site of disturbed flow

**Fig. 3.**
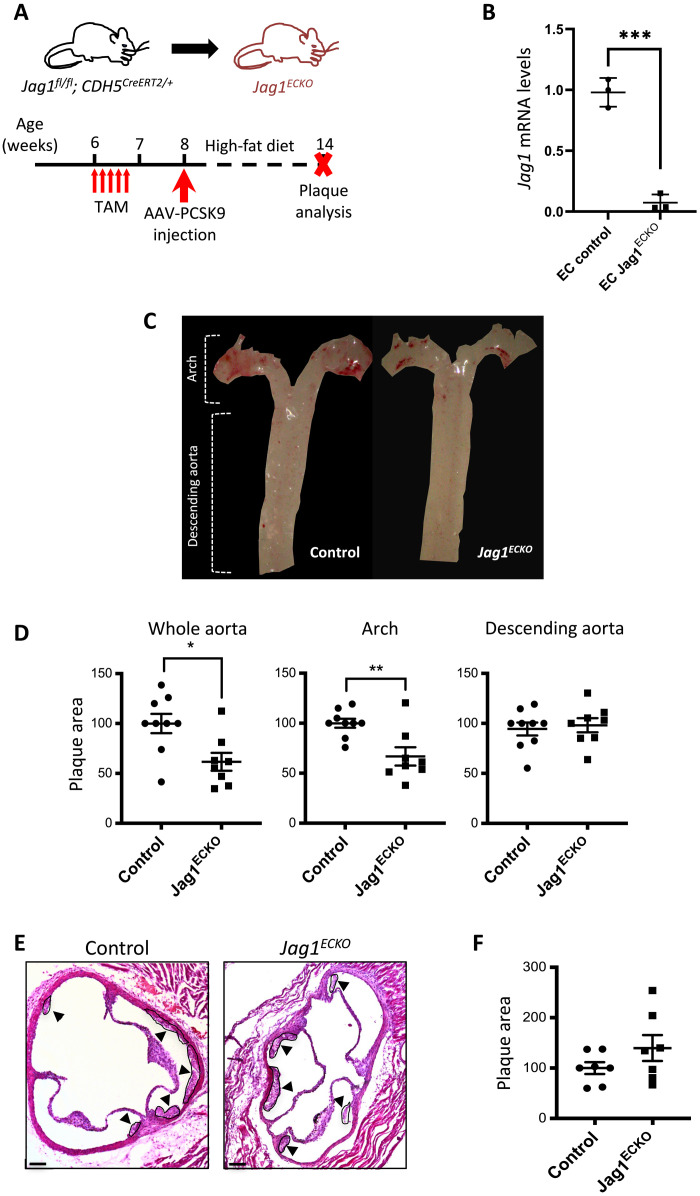
Loss of endothelial *Jag1* decreases plaque development in the aortic arch. (**A**) Timeline of *Jag1* deletion in a model of hypercholesterolemia. *Jag1^fl/fl^ CDH5^CreERT2/+^* (*Jag1^ECKO^*) mice aged 6 weeks and littermates lacking Cre (*Jag1^fl/fl^ CDH5^+/+^*; controls) received five intraperitoneal injections of tamoxifen (TAM) and one injection of AAV-PCSK9 virus at specified time points. After 6 weeks fed with high-fat diet, the mice were culled, and plaque area was quantified. (**B**) Validation of *Jag1* deletion. *Jag1* RNA was quantified in aortae isolated from *Jag1^ECKO^* mice (*n* = 3) and littermate controls lacking Cre 2 weeks after tamoxifen by endothelial RNA extraction and qRT-PCR. (**C**) Representative images of aortas stained with Oil Red O. (**D**) Quantification of plaque burden in the whole aorta, arch, and descending aorta was determined by calculating the percentage of aortic surface area covered by plaque for *Jag1^ECKO^* mice (*n* = 8) and littermate controls (*n* = 9). Representative images with plaque indicated (arrowheads) (**E**) and quantification of plaque burden (**F**) in the aortic roots of controls and *Jag1^ECKO^* mice. In all graphs, each data point represents one mouse, and means ± SEM are shown. Differences between means were analyzed using an unpaired *t* test. Scale bars, 100 μm.

To delineate the role of endothelial *Jag1* in atherosclerosis, we generated EC-specific inducible knockout mice by crossing a *Jag1* floxed strain with a transgenic strain expressing *CDH5^CreERT2/+^*. Mice aged 6 weeks were treated with tamoxifen for five consecutive days to delete *Jag1* from EC or to generate experimental controls and were then treated with adeno-associated viral proprotein convertase subtilisin/kexin type 9 (AAV-PCSK9) and exposed to a high-fat diet for 6 weeks to generate hypercholesterolemia and atherosclerotic lesions (Fig. 3A). Tamoxifen treatment of *Jag1^fl/fl^ Cdh5^CreERT2/+^* mice (*Jag1^ECKO^*) induced the deletion of *Jag1*, which was validated by qRT-PCR of endothelial RNA extractions (Fig. 3B) and en face staining of the aorta (fig. S8). In hypercholesterolemic mice, lesion area in the whole aorta was significantly reduced in *Jag1^ECKO^* compared to controls (*Jag1^fl/fl^ Cdh5^+/+^*) (Fig. 3, C and D). This reduction was not related to alterations in plasma cholesterol levels or triglycerides, which were similar in the experimental and control groups (fig. S9). Further analysis revealed that *Jag1^ECKO^* caused a reduction in lesion area in the aortic arch but not in the descending aorta (Fig. 3D). Lesion area in the aortic root was also unaltered in *Jag1^ECKO^* compared to controls (Fig. 3, E and F). Therefore, *Jag1* promotes atherosclerosis at an anatomically distinct site with specific flow conditions.

### JAG1 suppresses endothelial subsets that control vascular repair

**Fig. 4.**
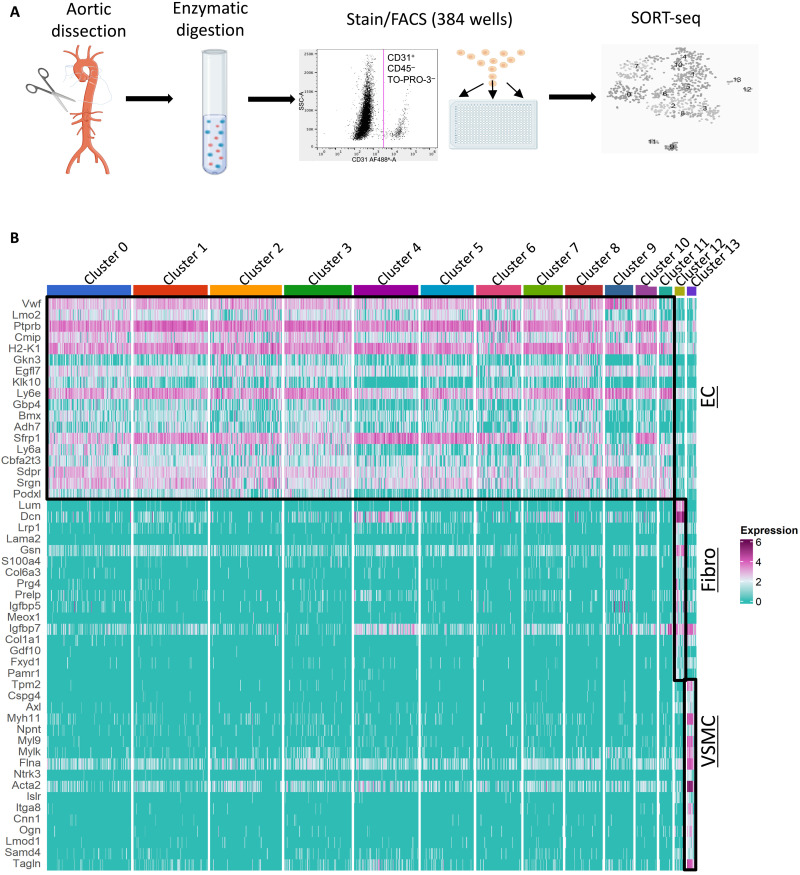
scRNA-seq identification of EC subsets. (**A**) Schematic overview of scRNA-seq workflow. Jag1^ECKO^ and control (Jag1^ECWT^) mouse aortas were dissected from the aortic root to the iliac artery and enzymatically digested to generate single-cell suspensions. After antibody staining, CD31^+^, CD45^−^, and TO-PRO-3^−^ cells were sorted into 384-well plates using FACS. scRNA-seq libraries were generated and sequenced using the SORT-seq method. (**B**) Aortas from *Jag1^ECKO^* and control mice were analyzed by FACS of CD31^+^ CD45^−^ cells coupled to scRNA-seq. Heatmap showing the most highly differentially expressed genes for each cluster. Twelve distinct clusters (0 to 11) defined as ECs exhibit common expression of several markers including the canonical markers *Vwf* and *Cdh5*. Cluster 13 expresses several canonical VSMC markers including *Myh11*, *Acta2*, and *Cnn1* and were therefore defined as VSMCs. Cluster 12 cells present higher expression of collagens/collagen-binding proteins (*Col1a1*, *Dcn*, and *Lum*), as well as reduced expression of VSMC-related proteins (*Myh11* and *Cnn1*), and were therefore defined as fibroblasts. Fibro, fibroblasts.

To elucidate the potential mechanisms of JAG1-dependent atherosclerosis, we performed scRNA-seq from control and *Jag1^ECKO^* aortas. Single cells were isolated from mouse aortas by enzymatic digestion, and CD31^+^ CD45^−^ TO-PRO-3^−^ ECs were purified by fluorescence-activated cell sorting (FACS) and processed for scRNA-seq (Fig. 4A). The scRNA-seq profiles of 991 cells from four experiments passed stringent quality control with >75% of the cells analyzed (fig. S10). On average, 1670 genes were detected per cell. Our SORT-seq analysis identified 14 distinct clusters (Fig. 4B). Of these, clusters 0 to 11 consistently expressed multiple EC markers, whereas cluster 12 contained fibroblast-like cells, and cluster 13 contained vascular smooth muscle cell (VSMC)–like cells (Fig. 4B). Clusters 12 and 13 were subsequently removed from further analysis of EC heterogeneity. Consistent with this, clusters 0 to 11 are closely similar to previously published EC clusters from the murine aorta (fig. S11) (*27*, *28*). Clusters 0 to 8 and 10 contain canonical EC markers, while both clusters 9 and 11 express markers that are strongly involved in angiogenesis and lipid storage, but cluster 9 also expresses markers that are characteristic of lymphatic endothelium (fig. S11). These observations confirm the presence of heterogeneity in normal mouse aortas.

**Fig. 5.**
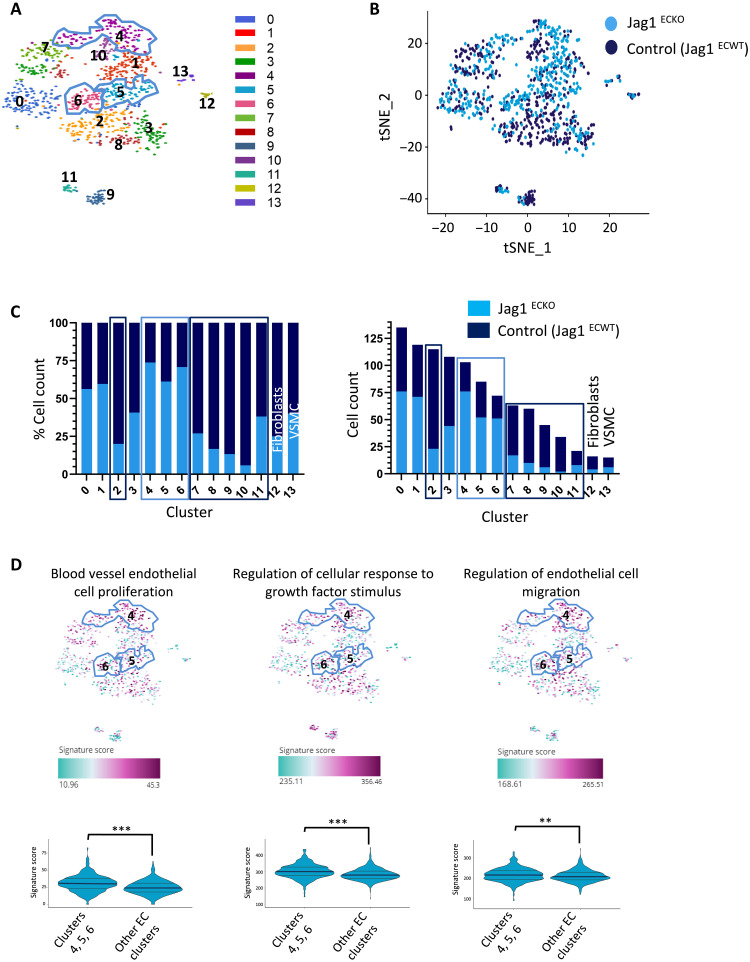
scRNA-seq analysis of *Jag1*-regulated endothelial heterogeneity. Aortas from *Jag1^ECKO^* and control mice were analyzed by FACS of CD31^+^ CD45^−^ cells coupled to scRNA-seq. (**A**) t-SNE representation of single-cell transcriptomes from Jag1^ECKO^ and control mice colored by cluster assignment. Clusters were identified using unbiased hierarchical clustering. (**B**) t-SNE showing the cell contribution of Jag1^ECKO^ and control mice to each subpopulation. (**C**) Bar graph showing the cell distribution of Jag1^ECKO^ and control mice across cell clusters, by percentage (left) and number of cells (right). Clusters 4 to 6 are largely composed of ECs derived from Jag1^ECKO^ mice, whereas cluster 2 and 7 to 11 are mainly composed of ECs derived from control mice. (**D**) Representation of enriched GO pathways on the t-SNE map. An overall score summarizing the expression of genes in each GO pathway is calculated for every cell in the scRNA-seq dataset. Differences between means were calculated using a Wilcoxon signed-rank test.

Most cells in clusters 4 to 6 were from *Jag1^ECKO^* mice, whereas clusters 2 and 7 to 11 contained cells mainly from control mice (*Jag1^ECWT^*; Fig. 5, A to C), indicating that Jag1 has a profound influence on EC heterogeneity. Cluster-specific nested functional enrichment of gene ontology (GO) terms revealed diverse functions associated with JAG1-regulated clusters (fig. S12). In particular, multiple GO terms relating to cell proliferation and migration were enriched in all three of the clusters enhanced in *Jag1^ECKO^* mice (clusters 4 to 6; Fig. 5D and fig. S12), suggesting that *Jag1* suppresses EC subtypes involved in proliferation and migration.

**Fig. 6.**
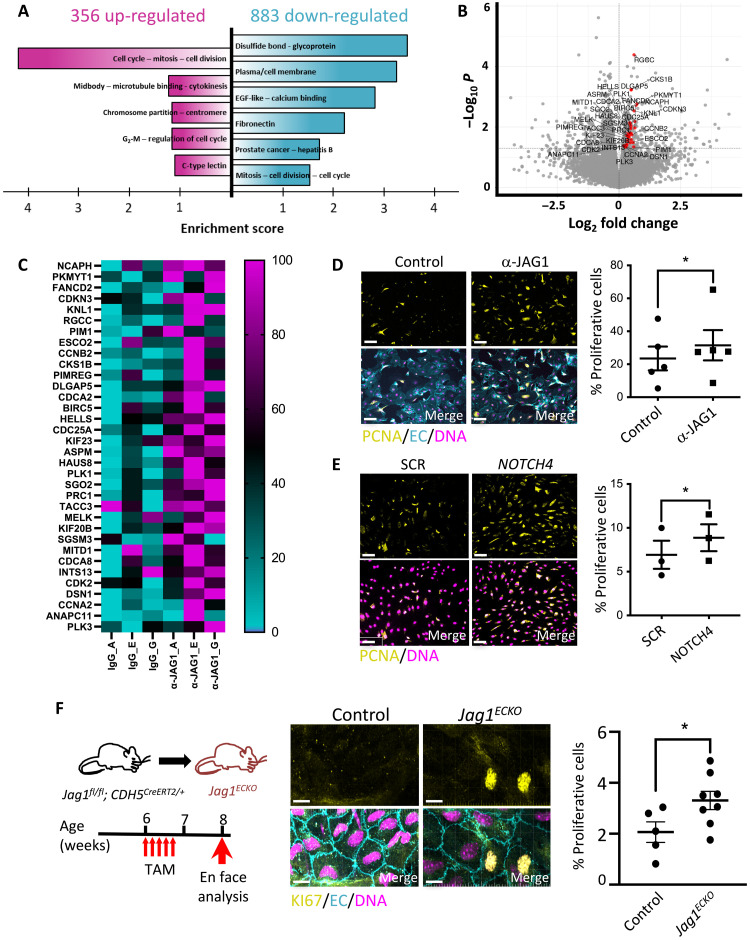
JAG1 activation by LOSS leads to inhibition of endothelial repair. (**A** to **C**) HCAECs were exposed to LOSS and treated with α-JAG1 blocking antibody or control isotype-matched antibodies for 48 hours before bulk RNA-seq. (A) Functional annotation clustering of the genes up-regulated (in magenta) and down-regulated (in cyan) in response to α-JAG1 (*P* < 0.05) using DAVID. Clusters with the highest enrichment score are shown. EGF, epidermal growth factor. (B) Genes associated with cell cycle are up-regulated following blockade with α-JAG1. Volcano plot displaying differentially expressed genes between JAG1 blockade and control samples. The *y* axis is the mean expression value of −log_10_ (*P* value), and the *x* axis displays the log_2_ fold change value. Significantly differentially expressed genes with a functional enrichment for the cell cycle are labeled and highlighted in red. (C) Heatmap showing the expression of proliferation-associated genes. Gene expression levels were normalized so that the highest expression is set to 100% and the lowest expression to 0 (*n* = 3). HCAECs were treated with α-JAG1 blocking antibody or control isotype-matched antibodies (*n* = 5) (**D**) or were treated with *NOTCH4* siRNA or with scrambled nontargeting sequences (SCR; *n* = 3) (**E**). Cultures were exposed to LOSS for 72 hours, and proliferation was quantified by immunofluorescence staining using antibodies against proliferating cell nuclear antigen (PCNA) (yellow). Differences between means were analyzed using paired *t* tests. Scale bars, 50 μm. (**F**) EC proliferation at an LOSS region of the aorta (inner curvature of arch) was quantified in control (*n* = 5) versus *Jag1^ECKO^* (*n* = 8) mice 2 weeks after tamoxifen injection by en face immunostaining using antibodies against Ki67 (yellow). Endothelium was costained (anti-CDH5; EC; cyan), and nuclei were detected using 4′,6-diamidino-2-phenylindole (DNA; magenta). Representative images are shown. The proportion of proliferative Ki67-positive cells (center) and the average total cell count per field of view (right) were calculated. Differences between means were analyzed using unpaired *t* test. Scale bars, 10 μm.

We next analyzed JAG1 function in HCAEC exposed to LOSS in vitro. Bulk RNA-seq revealed that 859 genes were significantly altered by inhibition of JAG1, and functional annotation using Database for Annotation, Visualization and Integrated Discovery (DAVID) found that the most highly enriched GO terms centered around cell division processes and cytokinesis/migration (Fig. 6, A to C). Thus, the combination of bulk RNA-seq analysis of cultured human cellular models and scRNA-seq analysis of murine EC identified JAG1 as regulator of EC proliferation. We substantiated this link by demonstrating that HCAEC proliferation and migration under LOSS are significantly enhanced by inhibition of the JAG1-NOTCH4 pathway using either gene silencing or activity-blocking antibodies (Fig. 6, D and E, and figs. S13, S14A, and S15). Moreover, the pan-Notch inhibitor *N*-[*N*-(3,5-difluorophenacetyl)-l-alanyl]-*S*-phenylglycine *t*-butyl ester(DAPT) enhanced the proliferation of HCAEC exposed to LOSS (fig. S14B), and this correlated with reduced levels of JAG1, whereas DLL4 was unaltered (fig. S14C), thereby further substantiating the link between JAG1, Notch, and proliferation under disturbed flow. Similarly, deletion of *Jag1* caused increased EC proliferation at a region of the mouse aorta exposed to LOSS (Fig. 6F) but did not influence EC proliferation at a region exposed to HSS (fig. S16). We conclude that the JAG1-NOTCH4 pathway suppresses proliferation and migration under disturbed flow. To investigate whether the effects of JAG1 were cell autonomous, we analyzed whether *JAG1*-silenced cells (unlabeled) can influence the proliferation of cocultured cells that were labeled with CellTracker. Proliferation was significantly enhanced in both labeled bystander cells and unlabeled *JAG1* small interfering RNA (siRNA)–treated cells compared to controls (fig. S17), revealing that JAG1 drives proliferation via a non–cell-autonomous mechanism. This effect was not recapitulated using conditioned medium from JAG1-inhibited cultures, indicating that soluble factors are not sufficient and the mechanism likely involves cell-to-cell contact. In summary, JAG1-NOTCH4 sensing of LOSS enhances disease susceptibility by altering EC heterogeneity to suppress EC subsets that control proliferation and migration.

## DISCUSSION

### JAG1/NOTCH4 mechanosensing in focal atherosclerosis

There is intense interest in the pathways that sense and respond to mechanical shearing forces in arteries because they are fundamental determinants of atherosclerosis, which is a major cause of worldwide death through heart attack and stroke (*1*). Here, we report that JAG1/NOTCH4 is a mechanosensitive pathway that promotes atherogenesis by suppressing reparative EC subsets.

**Fig. 7.**
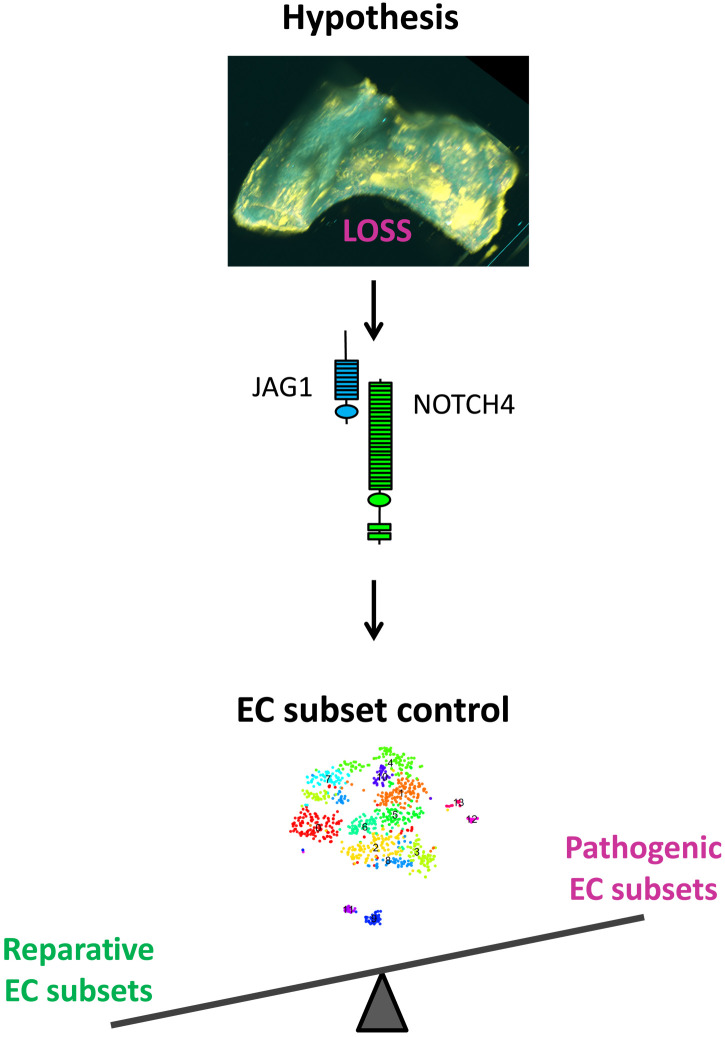
Hypothesis for JAG1-NOTCH4 control of reparative endothelial subsets in atherosclerosis. We hypothesize that LOSS activates JAG1-NOTCH4 signaling, which promotes atherosclerosis by altering the balance of reparative and pathogenic EC subsets.

Atherosclerosis develops at regions of arteries that are exposed to LOSS, whereas HSS regions are protected. Sensing of mechanical force is fundamental to the Notch signaling mechanism (*14*), and activation of Notch by shear stress is required for arterial differentiation (*15*, *29*, *30*), cardiac morphogenesis (*31*, *32*), and valve formation (*33*). Recent studies found that NOTCH1 is activated by HSS in adult arteries, and this pathway is a key regulator of adherens junctions and vascular permeability (*16*) and protects arteries from atherosclerosis (*17*). While physiological HSS is known to signal through NOTCH1 (*16*, *17*, *34*), here, we show that disease-causing LOSS signals via a JAG1-NOTCH4 pathway to promote atherosclerosis (Fig. 7). Our conclusion is based on the following observations: (i) JAG1 and NOTCH4 were exclusively enriched at LOSS regions of the aorta and were induced by LOSS in experimental flow–modified arteries, (ii) LOSS induces the expression of *NOTCH4* and *JAG1* and activates JAG1-NOTCH4 signaling in cultured EC, (iii) JAG1-NOTCH4 signaling represses EC subsets involved in vascular repair, and (iv) inducible deletion of *Jag1* from murine EC reduced atherosclerosis at the aortic arch that contains a LOSS region. It remains to be determined whether NOTCH4 signaling responds to LOSS directly, i.e., mechanical force transduction through the Notch receptor and/or ligand, or whether the response is indirect involving other mechanoreceptors (*35*), and further work is required to discriminate between these possibilities. We conclude that the Notch pathway is fundamental in controlling the spatial organization of atherosclerosis.

Our observations are consistent with previous findings that Notch receptors and ligands are expressed in plaques (*36*, *37*) and that global inhibition of Notch components can influence atherosclerosis in murine models (*36*, *38*, *39*). The function of Notch pathways varies considerably according to cellular context with tissue-specific deletion of Notch receptors and ligands, revealing roles in multiple vascular cell types including macrophages (*40*) and VSMCs (*41*). Here, we demonstrate that endothelial JAG1-NOTCH4 signaling is an essential driver of atherosclerosis specifically at sites of LOSS. The corollary is that therapeutic targeting of endothelial JAG1/NOTCH4 signaling in EC may provide a novel treatment strategy to prevent or treat atherosclerosis.

### Jag1 suppresses endothelial reparative subsets

To elucidate the mechanism of *Jag1*-dependent atherosclerosis, we carried out scRNA-seq in endothelial-specific *Jag1* knockout mice and found that Jag1 controls EC heterogeneity and represses endothelial subsets involved in proliferation and migration. Our study builds on previous reports of considerable EC heterogeneity in murine arteries (*27*, *28*, *42*, *43*) including the observations of Andueza *et al.* (*44*) who found that shear stress has a major effect on EC subsets. Here, we show that the Notch pathway, and in particular JAG1 signaling, is responsible for controlling EC subset plasticity. *Jag1* deletion had a major disruptive effect on EC heterogeneity by altering the prevalence of 9 of 11 EC subsets identified.

To further understand the mechanism of atherosclerosis, we next focused on three subsets that were enriched in *Jag1* endothelial knockouts and found that they were enriched for transcripts regulating proliferation and migration, suggesting that Jag1 represses EC involved in these processes. This observation was validated experimentally by demonstrating that JAG1 and *NOTCH4* are negative regulators of proliferation and migration in EC exposed to LOSS. It is accepted that EC proliferation is enhanced at sites of LOSS; however, this process has been implicated in atheroprotection (*45*) and atherogenesis (*46*, *47*). Thus, we propose that EC proliferation at the LOSS regions is a “double-edged sword.” Endothelium at the LOSS region is continually damaged by apoptosis, and therefore, proliferation is needed for vascular repair. Consistently, Schober *et al.* (*45*) showed that deletion of miR-126-5p, a microRNA that promotes cell proliferation, blocked EC repair in response to injury, and it also led to increased atherosclerotic development in miR126/ApoE double-knockout mice. Therefore, this study suggests that EC proliferation at low-shear regions is atheroprotective, as proliferative cells replace dead or lost cells after injury. Moreover, atherosclerosis at sites of LOSS is enhanced by senescence (irreversible cell cycle arrest) of EC, therefore implying that EC proliferation can have a protective role (*48*). On the other hand, excessive proliferation at LOSS regions can promote disease by increasing lipoprotein accumulation in arteries (*46*, *47*). The divergent effects of EC proliferation on atherogenesis and atheroprotection can be reconciled by the heterogeneous nature of arterial endothelium, i.e., some endothelial subsets may proliferate to repair vessels, whereas other subsets are characterized by excessive proliferation and lipid accumulation. We therefore hypothesize that JAG1 promotes atherosclerosis by suppressing specific EC subsets that are important for vascular repair (Fig. 7).

In summary, JAG1-NOTCH4 sensing of LOSS promotes atherosclerosis by altering EC heterogeneity, and we hypothesize that this pathway suppresses subsets that are responsible for vascular repair. These data demonstrate a fundamental role for JAG1-NOTCH4 in sensing disease-priming LOSS and suggest that therapeutic targeting of JAG1-NOTCH4 could be a novel treatment strategy to enhance reparative EC subsets to prevent or treat atherosclerosis.

### Limitations of the study

An inherent limitation of scRNA-seq is the loss of spatial information as the single cells are put into suspension; hence, further work is needed to understand the location of EC subsets in relation to shear stress. Cultured human cells and murine arteries do not exactly replicate the physiology of human arteries, and therefore, further work is required to translate these observations to human disease.

## MATERIALS AND METHODS

### Experimental design

Mice were bred so that *Jag1* could be deleted conditionally in EC of adults. This system was used to analyze the effects of *Jag1* deletion on (i) EC physiology assessed by scRNA-seq and en face staining coupled to confocal microscopy and (ii) atherosclerotic lesion formation measured by Oil Red O staining in hyperlipidemic mice. The expression pattern of Notch ligands and receptors was measured at HSS and LOSS regions of porcine arteries by qRT-PCR. The expression pattern of JAG1 and NOTCH4 was analyzed in diseased and healthy mouse arteries by light-sheet microscopy and by en face staining coupled to confocal microscopy. To assess directly whether flow regulates the Notch pathway, human ECs were cultured, and flow was applied using a pump before measurement of Notch components by qRT-PCR and immunoblotting. The function of JAG1 and NOTCH4 in cultured EC was analyzed by inhibiting them using either blocking antibodies or siRNA.

### Mice

Mice with inducible deletion of *Jag1* in EC were generated by crossing *Jag1^fl/fl^* (*49*) mice with *CDH5^Cre-ERT2^* mice (*50*). All mice were on a C57BL/6J background. PCR primers used for genotyping are described in table S1. To activate Cre, tamoxifen (Sigma-Aldrich) in corn oil was administered intraperitoneally for five consecutive days (2 mg per mouse per day). Two weeks after the first injection of tamoxifen, hypercholesterolemia was induced by intraperitoneal injection of adeno-associated virus containing a gain-of-function mutated version of the proprotein convertase subtilisin/kexin type 9 (rAAV8-D377Y-mPCSK9) gene (Vector Core, North Carolina), followed by a high-fat diet (829100, SDS UK) for 6 weeks as previously described(*51*). Constrictive cuffs were applied to the right carotid artery of anesthetized C57BL/6 mice as described previously (*22*). C57BL/6 and transgenic mice were housed under specific pathogen–free conditions. Animal care and experimental procedures were carried out under licenses issued by the U.K. Home Office, and local ethical committee approval was obtained. All animal procedures conformed to the guidelines from Directive 2010/63/EU of the European Parliament on the protection of animals used for scientific purposes and to Institutional Animal Care and Use Committee guidelines. Male mice between 1.5 and 3 months of age were used for experimentation.

### Endothelial RNA extraction

RNA was extracted from aortic EC using the QIAzol flushing method as described (*52*).

### Atherosclerosis plaque analysis

Mice were euthanized and perfused-fixed with phosphate-buffered saline (PBS), followed by 4% paraformaldehyde (PFA). The aorta was dissected, gently cleaned of adventitial tissue, and stained with Oil Red O (Sigma-Aldrich). The surface lesion area was analyzed using NIS elements analysis software (Nikon, NY). For analysis of aortic root sections, the upper portion of the hearts was dissected horizontally at the level of the atria and placed in 30% sucrose for 24 hours before embedding in optical cutting temperature. Serial 7-μm sections were processed for staining with Mayer’s hematoxylin. NIS elements analysis software (Nikon, NY) was used to calculate the total lesion area.

### Plasma lipid measurements

Blood samples were collected by terminal cardiac puncture, and plasma was separated by centrifugation for further analysis using a cobas analyzer (total plasma cholesterol, non–high-density lipoprotein cholesterol, and triglycerides).

### En face staining of murine endothelium

The expression levels of specific proteins were assessed in EC at regions of the inner curvature (LOSS site) and outer curvature (HSS site) of murine aortae, descending aorta (HSS site), or in carotid arteries by en face staining. Animals were euthanized by intraperitoneal injection of pentobarbital, and aortae were perfused in situ with PBS and then perfusion-fixed with 4% PFA before harvesting. Fixed aortae were tested by immunostaining using specific primary antibodies (table S2). ECs were identified by costaining using anti-CDH5 or anti-CD31 antibodies. Nuclei were identified using TO-PRO-3. Stained vessels were mounted before visualization of endothelial surfaces en face using confocal microscopy (Olympus SZ1000 confocal inverted microscope). The expression of particular proteins at each site was assessed by quantification of the mean fluorescence intensities with SEM.

### Tissue clearing and light-sheet microscopy

Murine aortas were optically cleared using the CUBIC method adapted from Susaki *et al.* (*53*). Samples were immersed in 50% CUBIC-1 solution (25% urea, 25% Quadrol, and 15% of Triton X-100) for 3 hours and then in fresh 50% CUBIC-1 solution for 16 hours. Samples were then washed using PBS and incubated with 50% CUBIC-2 solution (25% urea, 50% sucrose, and 10% triethanolamine) for 16 hours. The cleared samples were incubated with specific primary antibodies (table S2), and ECs were identified by costaining using anti-CDH5 antibodies. Stained vessels were mounted in 10% agarose gel and analyzed by light-sheet microscopy (Zeiss Light-sheet Z.1).

### Tissue processing and FACS for scRNA-seq

The left ventricle of the murine heart was perfused with heparin sodium (20 U/ml) in PBS. Aortas from age- and sex-matched *Jag1^ECKO^* and control mice (*Jag1^fl/fl^* *Cdh5^+/+^*) were dissected from the aortic root to below the iliac artery. After removing fat and connective tissue, aortas were transferred into ice-cold PBS. To generate single-cell suspensions, the isolated aorta was incubated in collagenase type I (450 U/ml), collagenase type XI (125 U/ml), hyaluronidase type 1-s (60 U/ml), deoxyribonuclease I (60 U/ml), and elastase (0.5 mg/ml) in PBS for 10 min at 37°C to allow for the separation of the adventitia cell layer. After removing the adventitia, tissue was divided into 2-mm pieces and further incubated in the enzymatic solution for 1 hour at 37°C to produce a single-cell suspension. The cell suspension was then filtered through a 40-μm cell strainer, washed, and resuspended in 1% bovine serum albumin (BSA)–PBS. For the remainder of the procedure, cells were kept on ice. Single-cell suspensions were incubated for 5 to 10 min with TruStain FcX (anti-mouse CD16/32) antibody to block nonspecific binding of immunoglobulin to Fc receptors, before staining with allophycocyanin-conjugated anti-CD45 and Alexa Fluor 488–conjugated anti-CD31. To exclude dead cells, samples were stained with TO-PRO-3. Stained aortic cells were washed with 1% BSA-PBS buffer before analysis, and CD31^+^, CD45^−^, and TO-PRO-3^−^ cells were sorted into 384-well plates containing reverse transcription primers, deoxyribonucleotide triphosphates, External RNA Controls Consortium (ERCC) RNA Spike-Ins, and mineral oil using a BDFACSMelody cell sorter (BD Biosciences). Plates containing capture cells were immediately stored at −80°C before sequencing.

### Single-cell RNA sequencing

scRNA-seq libraries were generated using the SORT-seq protocol (*54*), a partially robotized method based on the CEL-Seq2 protocol (*55*). Briefly, single cells were lysed at 65°C for 5 min, and second-strand mixers and reverse transcriptase were then added to the wells using the Nanodrop II liquid handling platform (GC Biotech). After reverse-transcribing the mRNA of each cell, double-stranded complementary DNAs (cDNAs) from single cells were pooled, and in vitro transcription was performed for linear amplification, which resulted in amplified RNA. TruSeq small RNA primers (Illumina) were used to prepare the Illumina sequencing libraries, and these DNA libraries were then sequenced paired-end at 75–base pair read length using the Illumina NextSeq (performed commercially by Single Cell Discoveries, Utrecht, The Netherlands). Both the RNA yield of the amplified RNA and the quality and concentration of the final cDNA libraries were assessed by Bioanalyzer (Agilent).

### Bioinformatics

Reads from Illumina sequencing were aligned to the GRCm38 mouse genome using STAR. Single-cell transcriptomes generated using SORT-seq were filtered by requiring the total number of genes detected per cell to be more than 1200. The percentage of reads mapping to ERCC controls was below 35%. Data were not filtered according to mitochondrial reads, which were negligible (minimum, 0.3%; median, 0.9%; maximum, 11.0%). After filtering count data and selecting high-quality single cells, the data were normalized, and the top 2000 highly variable genes in our dataset were selected and used for principal components analysis. The first 12 principal components were retained on the basis of their contribution to overall variability and used for downstream analysis (dimensional reduction and clustering). Dimensional reduction and clustering of our scRNA-seq dataset were performed using the R package Seurat (v3.1). Differential expression analysis was performed between clusters, and marker genes for each cluster were determined with the Wilcoxon rank sum test with *P* < 0.001 and a minimum log fold change threshold of 0.25 using Seurat and BBrowser (version 2.10.10). Differential expression analysis, heatmaps of gene expression embedded on hierarchical clustering, t-distributed stochastic neighbor embedding (t-SNE) representations showing the expression of defined genesets/GO pathways, and expression of single transcripts on the t-SNE embedding were performed using the software BBrowser (version 2.10.10). t-SNE representation of the expression of defined GO pathways shows the signature score, which indicates the sum of all features in each GO pathway.

### Isolation of EC from porcine aortae

Pig aortas from 4- to 6-month-old animals were obtained immediately after slaughter from a local abattoir. They were cut longitudinally along the outer curvature to expose the lumen. ECs exposed to high (=outer curvature) or low (=inner curvature) wall shear stress (WSS) were harvested using collagenase (1 mg/ml for 10 min at room temperature) before gentle scraping.

### EC culture and exposure to WSS

HCAECs were purchased from PromoCell and cultured according to the manufacturer’s recommendations. Experiments were performed using cells from multiple donors that were not pooled. HCAECs at passages 3 to 5 were seeded onto gelatin-coated ibidi μ-Slides I^0.4^ (Luer ibiTreat, ibidi) and used when fully confluent. Flowing medium was then applied using the ibidi pump system to generate high (13 dyne/cm^2^) or low oscillatory WSS. For low oscillatory WSS, HCAECs were exposed to a repeated cycle of 2 hours of oscillatory flow (±4 dyne/cm^2^, 0.5 Hz), followed by 10 min of unidirectional flow (+4 dyne/cm^2^), to ensure redistribution of nutrients (*56*). The slides and pump apparatus were placed in a cell culture incubator at 37°C.Inhibition of Notch activity was performed by the addition of DAPT (50 μM; Calbiochem) or blocking antibodies for Notch ligands (10 μg/ml; anti-JAG1 or anti-DLL4) (table S2). Humanized phage antibody YW152F targeting DLL4 (*7*) was provided by Genentech.

### Gene silencing

HCAEC cultures were transfected with siRNA sequences that are known to silence *NOTCH4* (L-011883-00, Dharmacon) or *JAG1* (L-011060-00, Dharmacon) using the Lipofectamine RNAiMAX transfection system (13778-150, Invitrogen), following the manufacturer’s instructions. Nontargeting scrambled sequences were used as a control (D-001810-01-50 ON-TARGETplus Non-targeting siRNA#1, Dharmacon).

### Real-time PCR and RNA-seq

RNA was extracted using the RNeasy Mini Kit (74104, QIAGEN) and reverse-transcribed into cDNA using the iScript cDNA synthesis kit (1708891, Bio-Rad). qRT-PCR was used to assess the levels of transcripts with gene-specific primers (table S3). Reactions were prepared using SsoAdvanced Universal SYBR Green supermix (172-5271, Bio-Rad) and following the manufacturer’s instructions and were performed in triplicate. Expression values were normalized against the housekeeping gene (mouse *Tbp*, human *HPRT*, or porcine *B2M*). Data were pooled from at least three independent donors, and mean values were calculated with SEM. For RNA-seq, the purity and integrity of total RNA samples isolated from HCAEC were assessed using a Bioanalyzer (Agilent), and high-quality samples from HCAEC donors were used to prepare RNA-seq libraries that were sequenced on an Illumina HiSeq platform yielding 20 million reads per sample. Library preparation and cDNA sequencing were performed by Novogene. Fastq samples were processed using the RNA-seq pipeline implemented in the bcbio-nextgen project https://bcbio-nextgen.readthedocs.io/en/latest/. After quality control checking using fastQC, RNA-seq reads were aligned to the human reference genome (assembly GRCh37/hg19) using STAR (*57*) with the default parameters. FeatureCounts (*58*) was used to create a matrix of mapped reads per Ensembl annotated gene. Differential gene expression was performed using the DESeq2 R package (*59*). Functional enrichments for protein-coding genes with *P* < 0.05 and log_2_ fold change > 0 were calculated using DAVID (*60*) using the total genes present as a background set.

### Immunofluorescent staining of cultured EC

HCAECs were fixed with PFA (4%) and permeabilized with Triton X-100 (0.1%). Following blocking with goat serum for 30 min, monolayers were incubated for 16 hours with primary antibodies against proliferating cell nuclear antigen (PCNA; proliferation marker) and CDH5 (endothelial marker) (table S2) and Alexa Fluor 488– or Alexa Fluor 568–conjugated secondary antibodies. Nuclei were identified using 4′,6-diamidino-2-phenylindole (Sigma-Aldrich). Images were taken with a widefield fluorescence microscope (DMI4000B, Leica) and analyzed using ImageJ software (1.49p) to calculate the frequency of positive cells. Isotype controls or omission of the primary antibody was used to control for nonspecific staining.

### Immunoblotting

Total cell lysates were isolated using lysis buffer (containing 2% SDS, 10% glycerol, and 5% β-mercaptoethanol). Primary antibodies used and concentrations are described in table S2. Horseradish peroxidase–conjugated secondary antibodies (Dako) and chemiluminescent detection were carried out using ECL Prime (GE Healthcare). Membranes were imaged using the Gel Doc XR+ system (Bio-Rad).

### In vitro migration assay

HCAECs were cultured until confluent in six-well plates and exposed to LOSS using an orbital shaking platform housed inside a cell culture incubator and rotating at 210 rpm. This system generated LOSS (approximately 5 dyne/cm^2^) with rapid variations in direction at the center. After 3 days of culture under these conditions, wounds were created on confluent HCAEC monolayers using a pipette tip. Cell migration was monitored by brightfield microscopy, and the distance migrated and wound width (gap width) were calculated.

### Cell tracking

HCAECs were incubated with CellTracker Red CMTPX dye (Invitrogen, #C34552) for 30 min. The next morning, the cells were used for experimentation.

### Statistical analysis

Data are presented as mean values ± SEM. Statistical analysis was performed with GraphPad Prism software. The degree of significance is as follows: *P < 0.05; **P < 0.01; ***P < 0.001. The test performed is indicated in the figure legend.

## Notes

View/request a protocol for this paper from *Bio-protocol*.
