# Supplementary material for: JAG1-NOTCH4 mechanosensing drives atherosclerosis
Source: Sci Adv. 2022 Aug 31;8(35):eabo7958. doi: 10.1126/sciadv.abo7958 (PMC9432841; doi:10.1126/sciadv.abo7958)
Supplement: Supplementary file 1 — Figs. S1 to S17 Tables S1 to S3 [file sciadv.abo7958_sm.pdf]

Science Advances
AAAS

Supplementary Materials for

JAG1-NOTCH4 mechanosensing drives atherosclerosis

Celine Souilhol *et al.*

Corresponding author: Celine Souilhol, c.souilhol@sheffield.ac.uk;
Jovana Serbanovic-Canic, j.serbanovic-canic@sheffield.ac.uk; Paul C. Evans, paul.evans@sheffield.ac.uk

Sci. Adv. **8**, eabo7958 (2022)
DOI: 10.1126/sciadv.abo7958

This PDF file includes:

Figs. S1 to S17
Tables S1 to S3

Fig. S1.

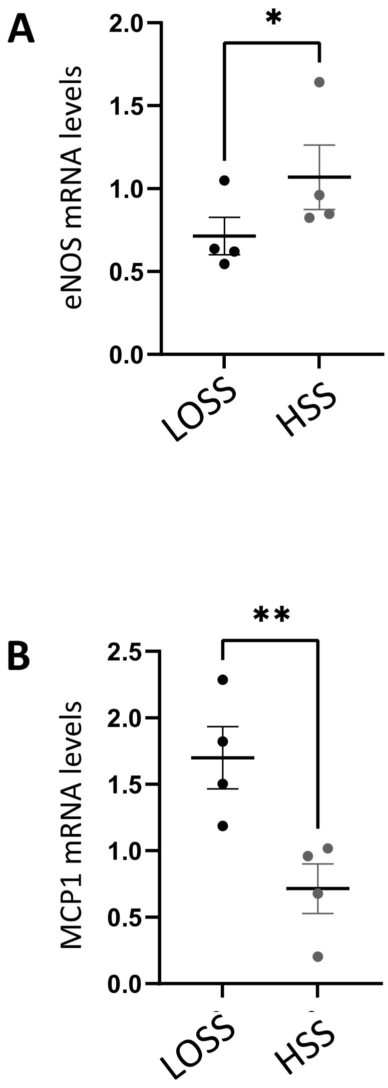

Validation of porcine aortic endothelial samples.

Cells were isolated from LOSS versus HSS regions in the porcine aortic arch. The levels of *eNOS* (A) and *MCP1* (B) were quantified in each population by qRT-PCR (n=4). Differences were analysed using paired *t*-tests.

Fig. S2.

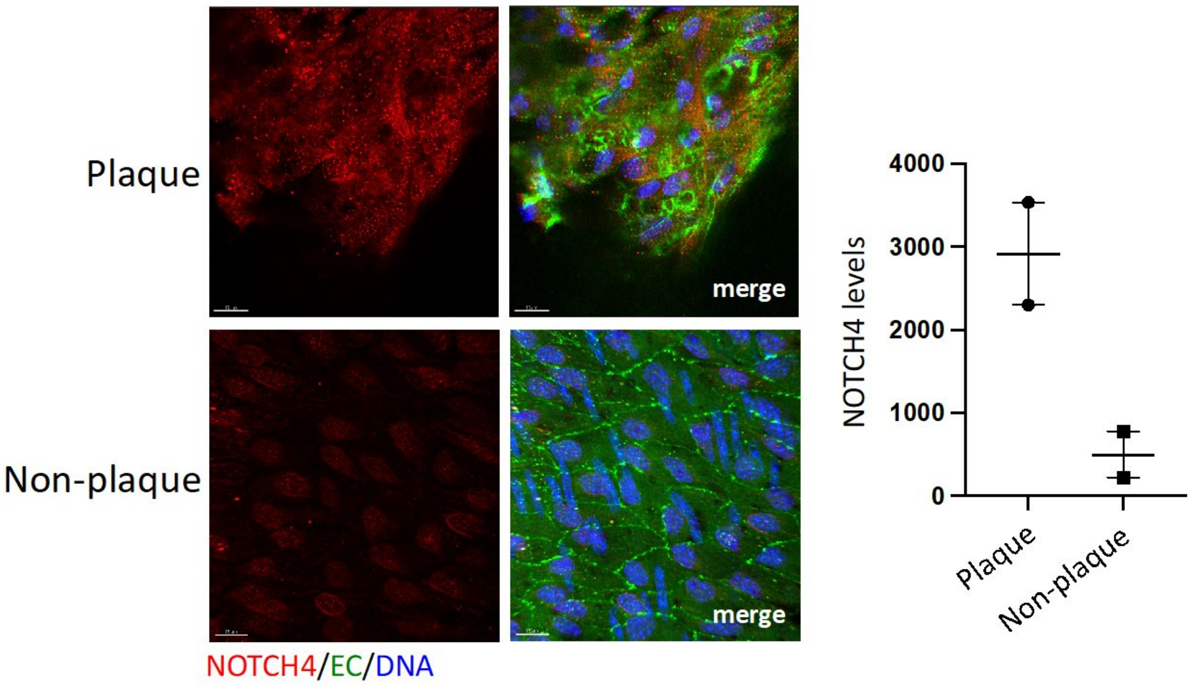

Plaque endothelial cells express NOTCH4.

Aortic arches were isolated from $ApoE^{-/-}$ mice exposed to a high fat diet for 6 weeks and *en face* immunostaining was performed using anti-NOTCH4 antibodies (red). Endothelium was co-stained (anti-CDH5; EC; green) and nuclei detected using TO-PRO-3 (DNA; blue). Representative confocal microscopy images of plaque areas and non-plaque areas are shown. The graphs on the right represent the red mean fluorescence intensity (MFI) (N=2).

Fig. S3.

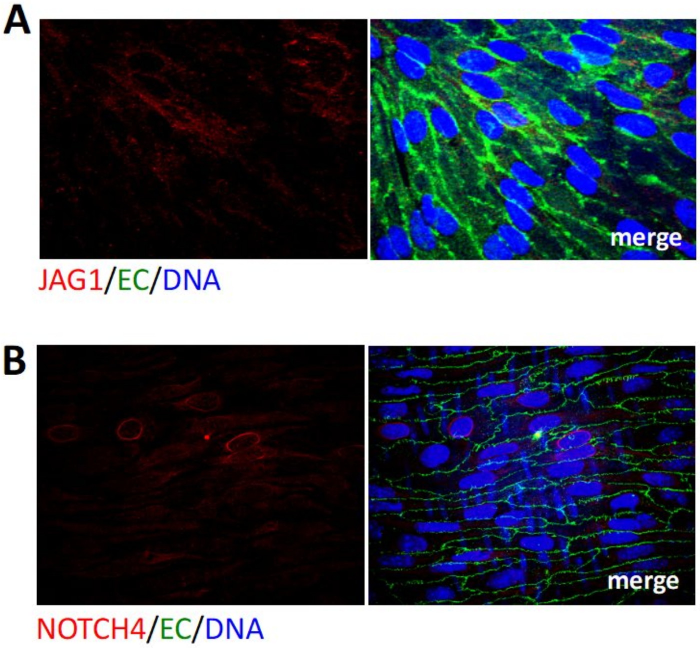

Low JAG1 and NOTCH4 expression in unmodified contralateral carotid arteries of mice. Flow-altering, constrictive cuffs were placed on the right carotid arteries of C57BL/6 mice; contralateral left carotid arteries were unmodified. Carotid arteries were harvested after 14 days, and *en face* staining was performed using anti-JAG1 (A) or anti-NOTCH4 (B) antibodies (red). Endothelium was co-stained (anti-CDH5; EC; green) and nuclei detected using TO-PRO-3 (DNA; blue). Representative images show low expression of JAG1 and NOTCH4 in contralateral carotid arteries.

Fig. S4.

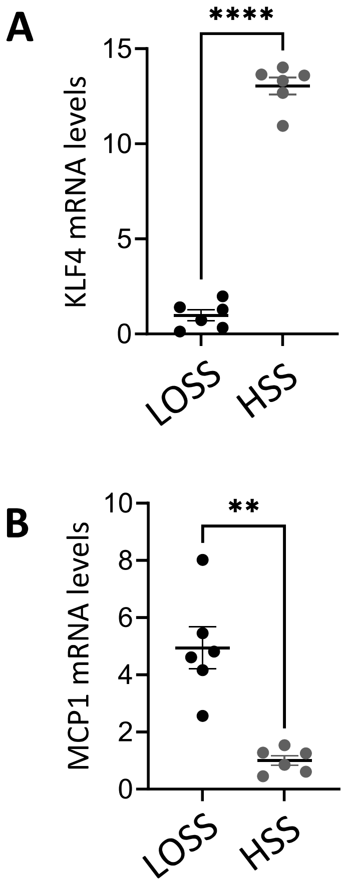

Validation of HCAEC responses to flow.

HCAEC were seeded on µ-slides and cultured under LOSS or HSS for 72h using the Ibidi system. Expression level of the known shear-sensitive genes *KLF4* (A) and *MCP-1* (B) was assessed by qRT-PCR. (n=6). Differences were analysed using paired *t*-tests.

Fig. S5.

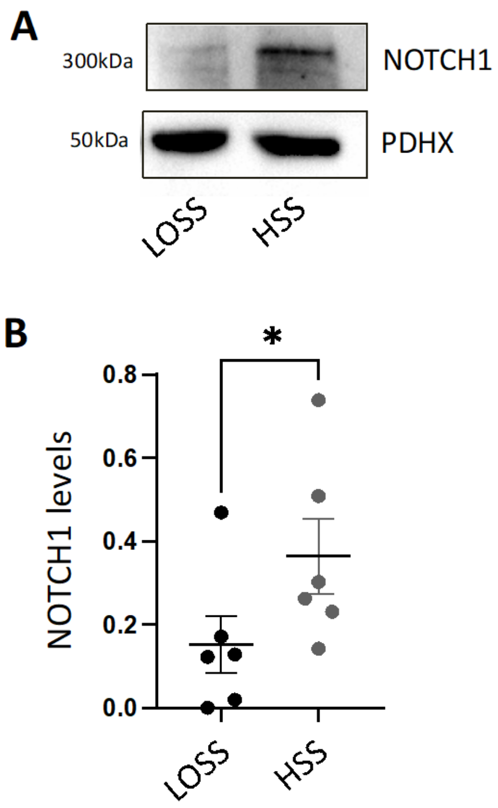

HSS induces NOTCH1 in arterial endothelial cells.
HCAEC were seeded on µ-slides and cultured under LOSS or HSS for 72h using the Ibidi system. Protein levels of NOTCH1 were quantified by immunoblotting. Representative images (A) and mean values normalized to the level of PDHX (n=6) (B) are shown. Differences between means were analysed using a paired t-test.

Fig. S6.

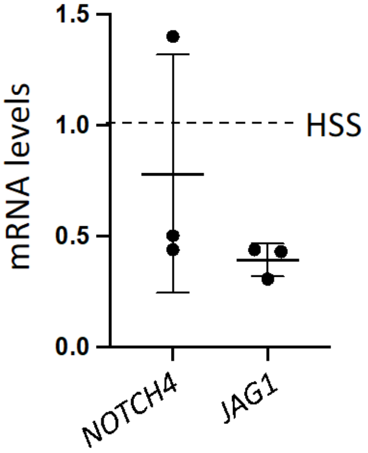

HCAEC express *JAG1* and *NOTCH4* mRNA levels at low levels in static conditions. HCAEC from individual donors were seeded on µ-slides and cultured under static conditions or under HSS for 72h using the Ibidi system. Levels of NOTCH4 and JAG1 mRNA were quantified by qRT-PCR. (n=3). Data points and mean +/- SEM from static HCAEC are shown, and mean levels under HSS are represented as a broken line.

Fig. S7.

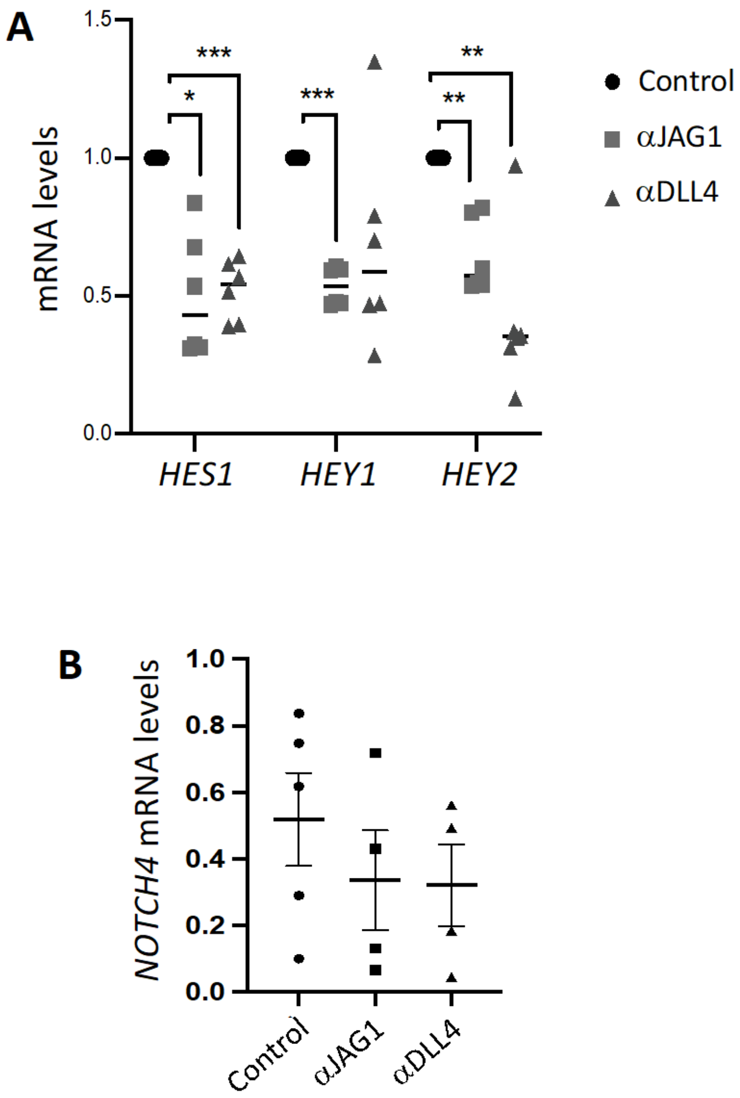

Validation of JAG1 and DLL4 blocking antibodies and effect of *NOTCH4*.
HCAEC were cultured under LOSS for 48h in the presence or absence of blocking antibodies against JAG1 or DLL4 (10 µg/ml). (A) Expression levels of Notch target genes (*HES1, HEY1, HEY2*) were quantified by qRT-PCR. (n=5-6). (B) Expression levels of *NOTCH4* were quantified by qRT-PCR. (n=4-5). Differences between means were analysed using one-way ANOVA.

Fig. S8.

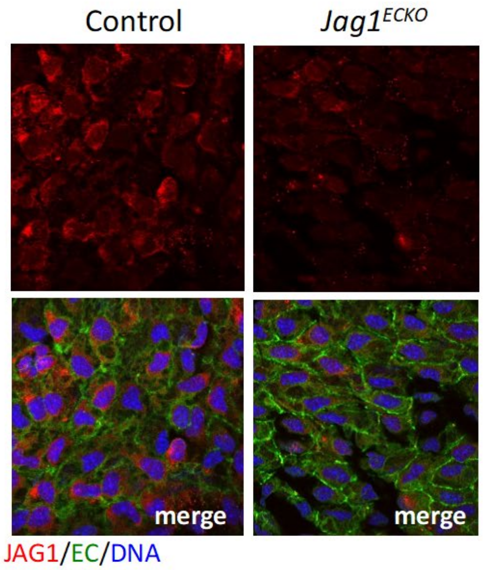

Validation of *Jag1* deletion.
Jag1^{ECKO} and control mice were analysed 2 weeks post-tamoxifen (TAM) injection. The expression of JAG1 protein (red) was visualized in the murine aorta by en face staining. Endothelium was co-stained (anti-CD31; EC; green) and nuclei detected using TO-PRO-3 (DNA; blue).

Fig. S9.

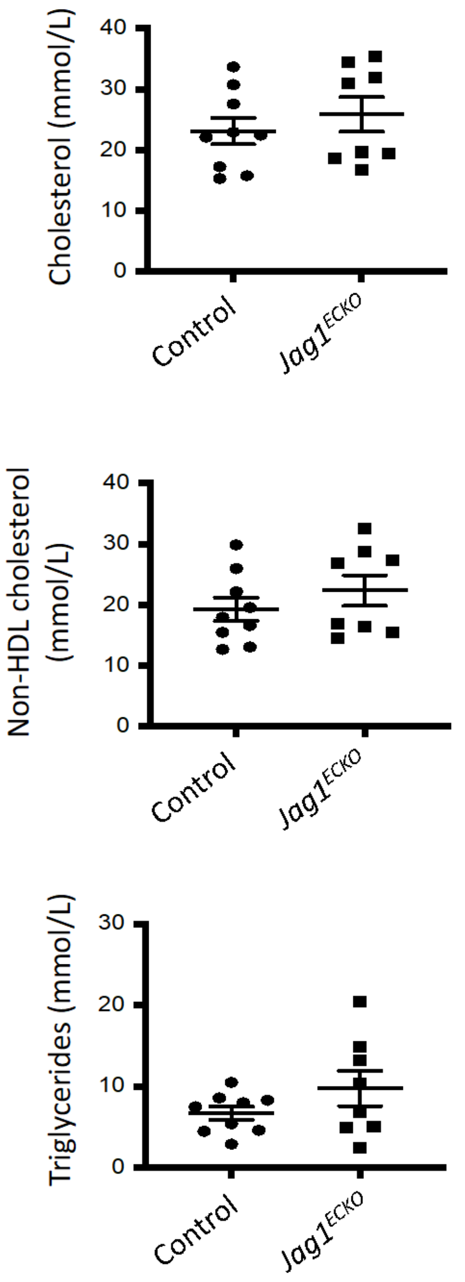

Lipid profiles in *Jag1*^{ECKO} mice.

Jag1^{ECKO} mice aged 6 weeks and littermate controls received five intraperitoneal injections of tamoxifen and one injection of PCSK9-AAV virus at specified time points. After 6 weeks fed with high fat diet, total cholesterol, non-HDL cholesterol and triglyceride levels were measured in *Jag1*^{ECKO} mice and controls. Differences between means were analyzed using an unpaired *t*-test.

Fig. S10.

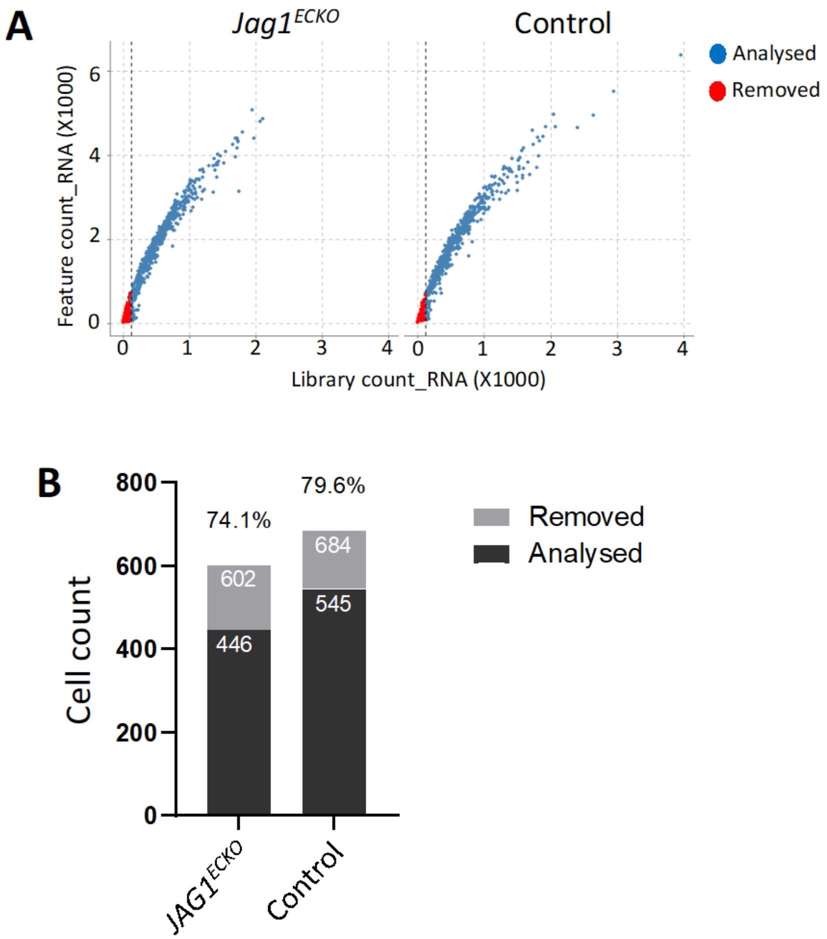

scRNAseq analysis quality control (QC).

Aortas from *Jag1*^{ECKO} and control mice were analysed by FACS of $CD31^{+}$ $CD45^{-}$ cells coupled to scRNAseq. (A) Scatter plot showing Library Count- Feature Count relationship per genotype. Library count is the total number of counts for each cell and Feature Count is the number of features for the cells that have detected expression. Cells are shown as *Removed* (cells filter by having a "Feature Count" less than 1200); and Analyzed (cells used in the subsequent analysis). (B) Bar graph showing remaining cells by genotype after QC analysis. Initial numbers of cells are represented in grey whereas remaining cells after QC filtering are represented in black. Percentage of cells by genotype used for subsequent analysis is shown at the top of each bar.

Fig. S11.

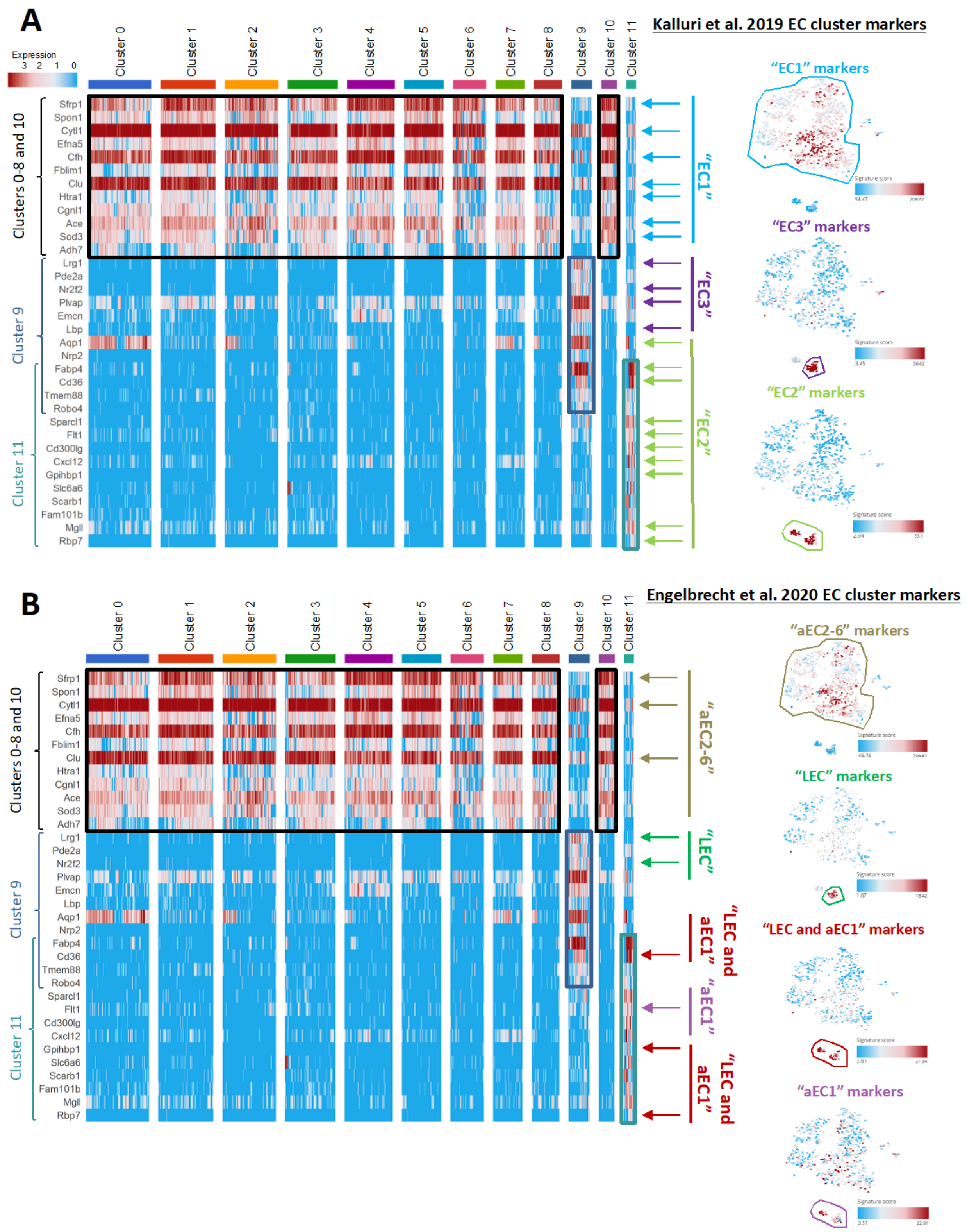
A
Expression
3 2 1 0
Cluster 0
Cluster 1
Cluster 2
Cluster 3
Cluster 4
Cluster 5
Cluster 6
Cluster 7
Cluster 8
Cluster 9
Cluster 10
Cluster 11
Kalluri et al. 2019 EC cluster markers
Clusters 0-8 and 10
Cluster 9
Cluster 11
Sfrp1
Spon1
Cytl1
Efna5
Cfh
Fblim1
Clu
Htra1
Cgnl1
Ace
Sod3
Adh7
Lrg1
Pde2a
Nr2f2
Plvap
Emcn
Lbp
Aqp1
Nrp2
Fabp4
Cd36
Tmem88
Robo4
Sparcl1
Flt1
Cd300lg
Cxcl12
Gpihbp1
Slc6a6
Scarb1
Fam101b
Mgll
Rbp7
"EC1"
"EC3"
"EC2"
"EC1" markers
"EC3" markers
"EC2" markers
Signature score
B
Engelbrecht et al. 2020 EC cluster markers
"aEC2-6"
"LEC"
"LEC and aEC1"
"aEC1"
"aEC2-6" markers
"LEC" markers
"LEC and aEC1" markers
"aEC1" markers

Expression of aortic endothelial cell cluster markers from previous publications.
Aortas from *Jag1*^{ECKO} and control mice were analysed by FACS of CD31^{+} CD45^{-} cells coupled to scRNAseq. Heatmap showing the highest differentially expressed genes in endothelial clusters 0-8 and 10 relative to other EC clusters, in cluster 11 relative to other EC clusters, and in cluster 9 relative to other EC clusters. (A) Expression of 'EC1', 'EC2', and 'EC3' marker genes from Kalluri et al. (*27*) in clusters 1-11. The arrows indicate enrichment in the specified clusters. On the right, expression of 'EC1', 'EC2', and 'EC3' marker genes are shown on the t-SNE embedding. (B) Expression of 'aEC1', 'LEC', 'LEC and aEC1' and 'aEC2-6' marker genes from Engelbrecht et al. (*28*) in clusters 1-11. The arrows indicate enrichment in the specified clusters. On the right, expression of 'aEC1', 'LEC', 'LEC and aEC1' and 'aEC2-6' marker genes are shown on the t-SNE embedding. The highest expressed markers of 'EC1' from Kalluri et al. (*27*) which represent canonical EC markers, and the highest expressed markers of 'aEC2-6' from Engelbrecht et al. (*28*), were highly expressed by clusters 0-8 and 10 in our data set, including *Sfrp1, Cytl1, Cfh and Clu*. Most 'EC2' markers from Kalluri et al. (*27*), which are involved in lipid transport and angiogenesis, are mainly expressed in cluster 11, but some are also highly expressed in cluster 9 (e.g. *Fabp4, Cd36*). 'aEC1' markers from Engelbrecht et al. (*28*), although enriched in cluster 11, are also expressed in cluster 9. 'EC3' markers from Kalluri et al. (*27*) which are characteristic of lymphatic endothelium and strongly resemble 'LEC' cluster from Engelbrecht et al (*28*) were only expressed in cluster 9 (*Nr2f2, Lrg1, Plvap*).

Fig. S12.

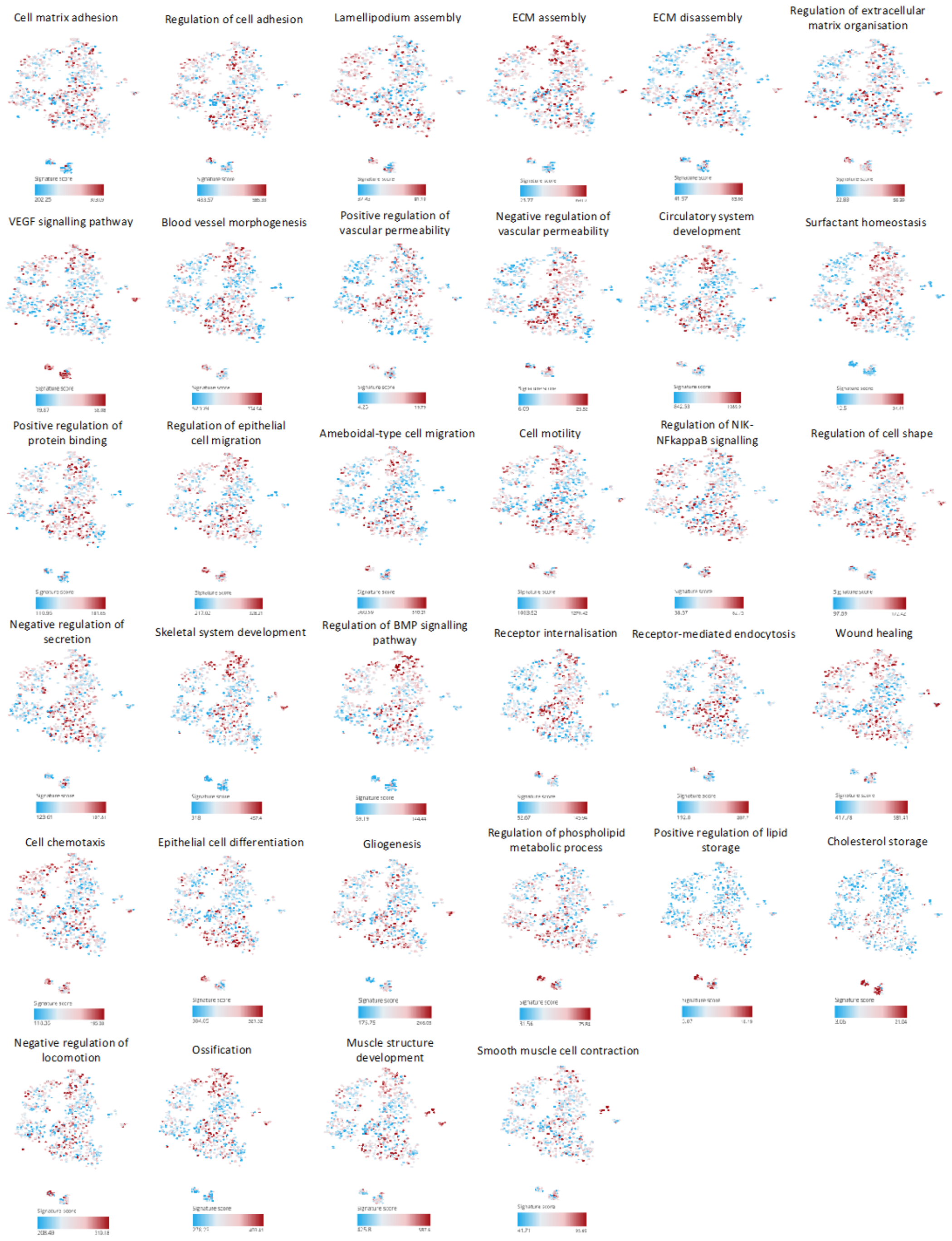

tSNE representation of GO terms for EC clusters.
Aortas from *Jag1*^{ECKO} and control mice were analysed by FACS of CD31^{+} CD45^{-} cells coupled to scRNAseq. tSNE representation of the scRNA-seq data showing the expression of defined gene

sets that determine different GO pathways. Signature score: sum of all features in each GO pathway.

Fig. S13.

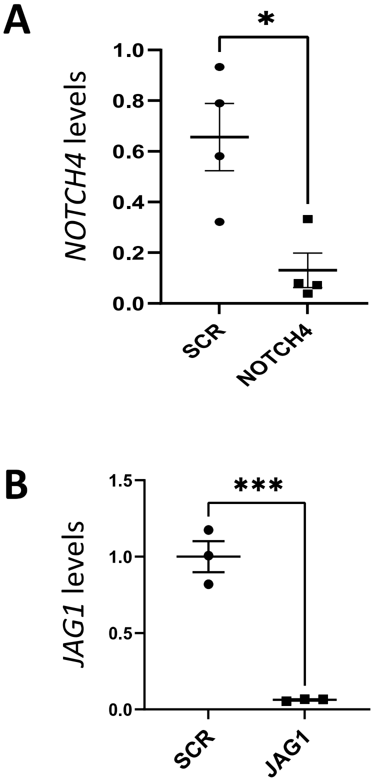

Validation of gene silencing.
HCAEC were treated with siRNA targeting *NOTCH4* (A) or *JAG1* (B) or with scrambled (SCR) control while exposed to LOSS for 48h using the Ibidi system. Expression levels of target genes were quantified by qRT-PCR (n=3-4). Differences between means were analysed using a paired *t*-test.

Fig. S14.

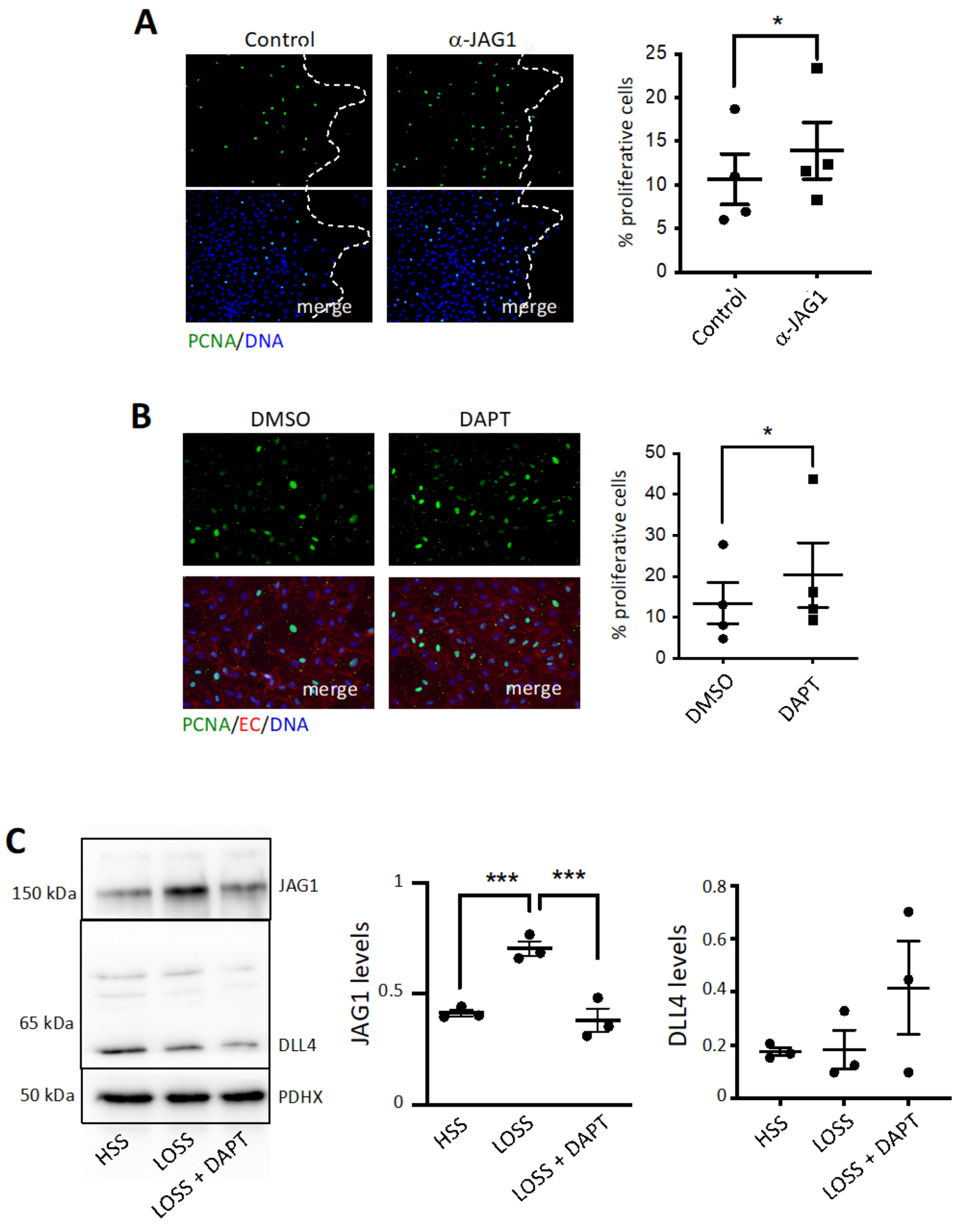

Notch signalling controls HCAEC proliferation under LOSS conditions.
(A) HCAEC were exposed to LOSS for 72h. To assess the role of JAG1 during endothelium repair, a scratch wound was made in the monolayer and the cells were treated with JAG1 blocking antibodies for 24h. Proliferation rate at the edge of the wound was then tested by using PCNA immunostaining (green). Nuclei were detected using DAPI (DNA; blue). (B, C) HCAEC were treated with either DAPT (γ-secretase inhibitor) or DMSO while exposed to LOSS or HSS for 72h using the Ibidi system. (B) Proliferation was quantified by immunofluorescence staining using antibodies against PCNA (green). Endothelium was co-stained (anti-CDH5; EC; red) and nuclei detected using DAPI (DNA; blue) (n=4). (C) Protein levels of JAG1 and DLL4 were quantified by immunoblotting and normalized to the level of PDHX (n=3). Differences between means were analysed using a paired t-test (A, B) or by ANOVA (C).

Fig. S15.

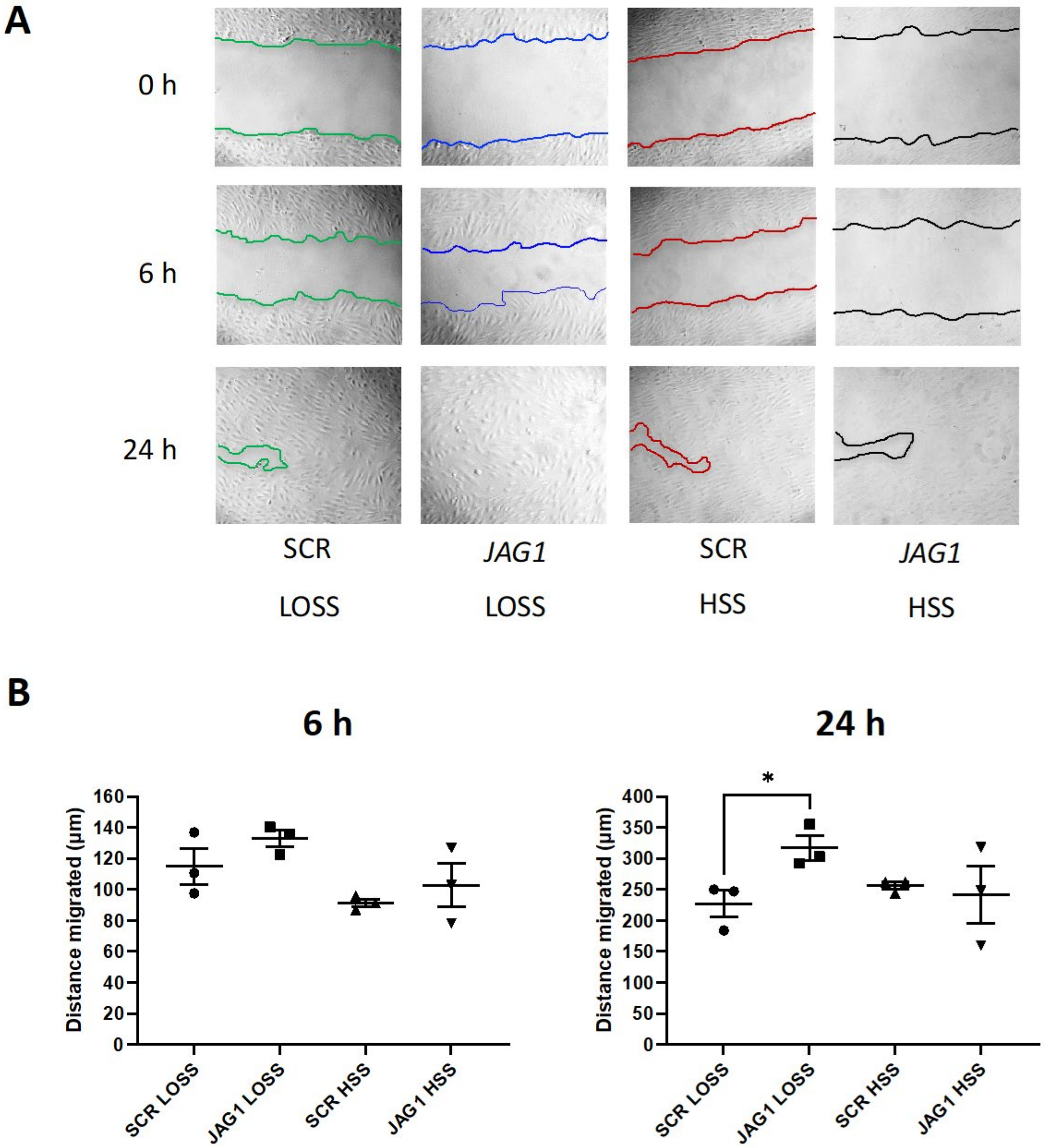

JAG1 reduces endothelial migration under LOSS conditions.

HCAEC were treated with siRNA targeting *JAG1* or with scrambled (SCR) control while exposed to LOSS or HSS for 48h using the orbital system. A scratch was then made and cell migration was monitored for 24h (n=3). (A) Representative images are shown with the leading edges indicated. (B) Distance migrated was calculated and mean values are plotted over time. Differences between means were analysed by ANOVA.

Fig. S16.

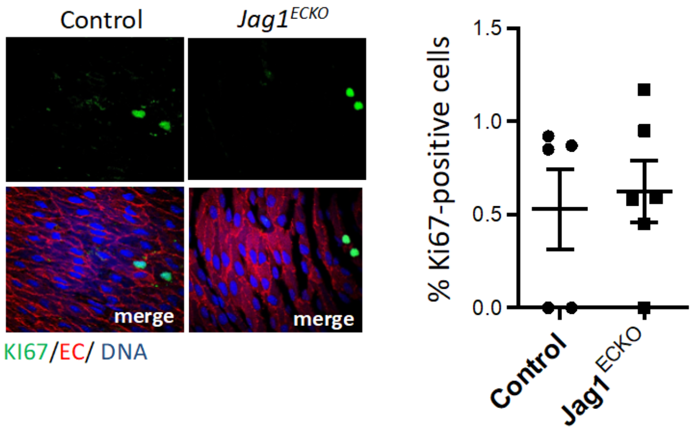

***Jag1* does not regulate endothelial proliferation at a HSS region of the aorta.**
Endothelial cell proliferation was quantified at the outer curvature of the aortic arch, a HSS region, in *Jag1*^{ECKO} (n=6) mice and control mice (n=5). Mice were analysed 2 weeks post-tamoxifen (TAM) injection by en face immunostaining of the aorta using antibodies against Ki67 (red). Endothelium was co-stained (anti-CD31; EC; red) and nuclei detected using TO-PRO-3 (DNA; blue). Representative images are shown. The proportion of proliferative Ki67-positive cells were calculated. Differences between means were analysed using an unpaired t-test.

Fig. S17.

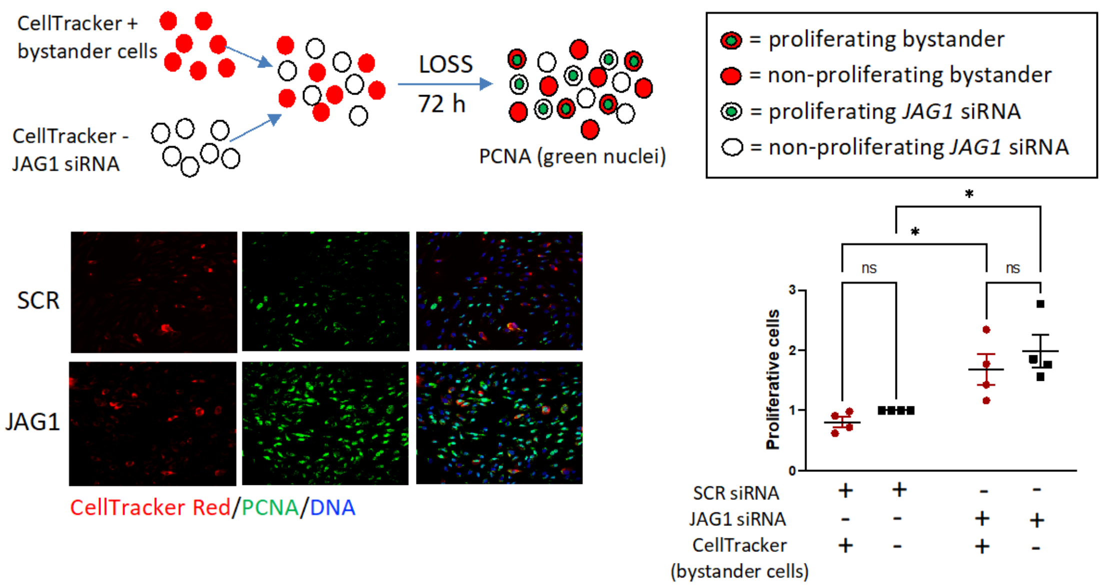

JAG1 controls proliferation in neighbouring endothelial cells.
Schematic diagram showing experimental setup (top). HCAECs transfected with *JAG1* siRNA or control (SCR) siRNA were mixed with an equal number of SCR siRNA-transfected cells labelled with CellTracker Red. The cells were exposed to LOSS for 72 hours and proliferation of labelled (CellTracker+) and non-labelled (CellTracker-) cells was assessed by PCNA staining (green) (bottom left) and nuclei were co-stained using DAPI (DNA). Quantification of proliferation (n=4) (bottom right). Differences between means were analysed by two-way ANOVA.

Table S1.

**Gene**	**Forward**	**Reverse**	**Size**
Jag1	GCA AGT CTG TCT GCT TTA TC	AGG TTG GCC ACC TCT AAA TC	Flox=317bp<br>WT=267bp
Cre	TCG ATG CAA CGA GTG ATG AG	AGT GCG TTC GAA CGC TAG AG	Cre=373bp

PCR primers for mouse genotyping.

Table S2.

Antibody	Company	Use	Final Concentration
**DLL4**	Genentech	Blocking antibody	10μg/ml
**DLL4**	Abcam (Ab7280)	WB<br>IF	0.2μg/ml<br>1μg/ml
**JAG1**	R&D systems	Blocking antibody	10μg/ml
**JAG1**	Santa Cruz (sc 6011)	WB<br>IF	0.2μg/ml<br>4μg/ml
**NOTCH1**	Cell Signaling Technology(3608)	WB	0.2μg/ml
**NOTCH4**	Santa Cruz (sc 5594)	WB<br>IF	1μg/ml<br>2μg/ml
**CDH5**	BD Bioscience (555661)	IF	1μg/ml
**CDH5**	BD Bioscience (555289)	IF	5μg/ml
**PCNA**	Abcam (ab 15497)	IF	5μg/ml
**Ki67**	Abcam (ab 15580)	IF	2μg/ml
**CD45**	Biolegend (103112)	FACS	2μg/ml
**CD31**	Biolegend (102514)	FACS	10μg/ml
**TruStain FcX™ CD16/32**	Biolegend (101320)	FACS	10μg/ml
**HEY1**	Proteintech (19929-1-AP)	WB	1μg/ml
**SMAD2/3**	R&D Systems (AF3797)	ChIP	5μg/ml

Antibodies used in the study. WB, Western blotting; IF, immunofluorescence

Table S3.

Porcine primers:

Gene	Forward	Reverse
NOTCH1	TGCCTGTGTCCACCTGGCTTCA	CTCCGTTTCGGCACAGGTGGGTA
NOTCH2	TCTGCTCACCAGGATTCA	CCTCGGGGCACATACAAC
NOTCH3	GCTCCTTGCCCCCACTCT	GAAACCCATTCCATCGCT
NOTCH4	TCCAAGAAATGCCCATAAAC	CACATAGTAGGTGCCCAATAAA
JAG1	TGTTAGCAAACGTGACGGGA	GGGGCACCAGGAAATCTGTT
JAG2	CTGGGTGGAGGATTGCAAC	CCCACACCACACCTTGCT
DLL1	GGAGAGAGGCGAGAAAGTCT	CGGCAAACAGATGGGCTC
DLL3	ATACTGGGTCTCGCTTGCTG	AATCTGAGGACGGGCTTGG
DLL4	ATGCAAGAAGCGCAATGACC	CAGACAGGCTGTTCGCAGTA
B2M	TTCACTCCTAACGCTGTGGA	GTGGTCTCGATCCCACTTAAC
CD45	GTGATGAGTTACTGGAAACCA	CTGCCAGAAGTCACCAATGG
CDH5	GAAACACAAGATGCCCAGGG	AAATGTGTACCTGGTCTGGG
SMA	CGATGAAGGAGGGCTGGAACAGGG	CGTGACCACTGCCGAGCGTGAGAT
ENOS	CGCTACAACATTCTGGAGGA	ACTTTGGCCAGCTGGTAACT

Mouse primers:

Gene	Forward	Reverse
Jag1	GAGGCGTCCTCTGAAAAACA	ACCCAAGCCACTGTTAAGACA
Dll4	CGGGAACCTTCTCACTCAAC	TTGGATGATGATTTGGCTGA
Tbp	GGGGAGCTGTGATGTGAAGT	CCAGGAAATAATTCTGGCTCA
Hprt	AGTCCCAGCGTCGTGATTAG	TCTCGAGCAAGTCTTTCAGTCC

Human primers:

Gene	Forward	Reverse
NOTCH1	CGGGGCTAACAAAGATATGC	CACCTTGGCGGTCTCGTA
NOTCH2	TGGTGGCAGAACTGATCAAC	CTGCCCAGTGAAGAGCAGAT
NOTCH3	AGCTTGGGAAATCAGCCTTA	TCCTTGCTATCCTGCATGTC
NOTCH4	CCTCTCTGCAACCTTCCACT	GCCTCCATTGTGGCAAAG
JAG1	GGCAACACCTTCAACCTCA	GCCTCCACAAGCAACGTATAG
JAG2	GTGGATGAGATCAACGGGTATC	AACCCGATCACTTCCTGGC
DLL1	GGGAGCTGCACGGATCTC	CACAGGTCATGGCACTCAAT
DLL3	CCGGATGCACTCAACAACC	TCCAATCTACGGACGAGCTC
DLL4	CCAGGGACTCCATGTACCA	GAGCAGGGATGTCCAGGTAG
HES1	GCACAGAAAGTCATCAAAGCC	TTCCAGAATGTCCGCCTT
HEY1	AAAAGCCGAGATCCTGCAGA	GTGCGCGTCAAAGTAACCTT

HEY2	GGTAAAGGCTACTTTGACGCA	GTACCGCGCAACTTCTGTTA
MCP-1	GCAGAAGTGGGTTCAGGATT	TGGGTTGTGGAGTGAGTGTT
KLF4	TAGCTCGAGGCATTCCAAGC	CCCGTGTGTTTACGGTAGTG
HPRT	TTGGTCAGGCAGTATAATCC	GGGCATATCCTACAACAAC

qRT-PCR primers used in the study.
